# Stochastic neutral drifts seem prevalent in driving human virome assembly: Neutral, near-neutral and non-neutral theoretic analyses

**DOI:** 10.1016/j.csbj.2022.03.027

**Published:** 2022-03-30

**Authors:** Zhanshan (Sam) Ma, Jiandong Mei

**Affiliations:** a Computational Biology and Medical Ecology Lab, Kunming Institute of Zoology, Chinese Academy of Sciences, China; b Center for Excellence in Animal Genetics and Evolution, Chinese Academy of Sciences, China; c Department of Thoracic Surgery, West China Hospital, Sichuan University, Chengdu, China

**Keywords:** Virome assembly, Hubbell’s unified neutral theory of biodiversity (UNTB), Sloan’s near-neutral model (SNM), Multisite neutral model (MSN), Power of neutral tests (PNT), Type-I & Type-II errors, Normalized stochasticity ratio (NSR)

## Abstract

It is estimated that human body is inhabited by approximately 380 trillions of viruses, which exist in the form of viral communities and are collectively termed as human virome. How virome is assembled and what kind of forces maintain the composition and diversity of viral communities is still an open question. The question is of obvious importance because of its implications to human health and diseases. Here we address the question by harnessing the power of Hubbell’s unified neutral theory of biodiversity (UNTB) in terms of three neutral models including standard Hubbell’s neutral model (HNM), Sloan’s near-neutral model (SNM) and Harris et al. (2017) multi-site neutral model (MSN), further augmented by Ning et al. (2019) normalized stochasticity ratio (NSR) and Hammal et al. (2015) power analysis for the neutral test (PNT). With the five models applied to 179 virome samples, we aim to obtain robust findings given both Type-I and Type-II errors are addressed and possible alternative, non-neutral processes are detected. It was found that stochastic neutral drifts seem prevalent: approximately 65–92% at metacommunity/landscape scales and 67–80% at virus species scale. The non-neutral selection is approximately 26–28% at community scale and 23% at species scale. The false negative rate is about 2–3%, which suggested rather limited confounding effects of non-neutral process on neutrality tests. We postulate that prevalence of neutrality in human virome is likely due to extremely simple structure of viruses (stands of DNA/RNA) and their inter-species homogeneities, forming the foundation of species equivalence—the hallmark of neutral theory.

## Graphical abstract

**Figure:**
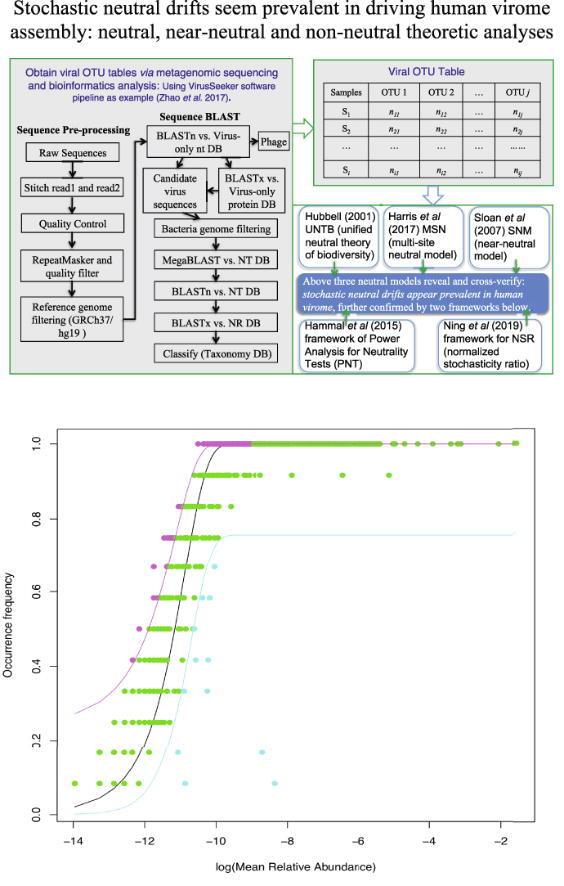

## 1 Introduction

Our body does not consist of our own cells alone; instead, it is cohabited by trillions of microorganisms, collectively termed as human microbiome. For this reason, some scientists consider our body as the *holobiont*, consisting of the host cells and all of its symbiotic microbes [1], [2]. It is estimated that the human body is inhabited by at least 38 trillion bacteria, which is about 10 times of the cell number of our body. However, the award for the most abundant microbes in the human microbiome cannot be awarded to bacteria, and instead the award goes to viruses which are estimated to exceed 380 trillions that are collectively termed as the human *virome* [3], [4]. Human virome is made of bacteriophages that predate bacteria and archaea, human-cell virus causing transient infections, endogenous retroviruses, as well as viruses leading to persistent and latent infections [5]. First principles of Darwin’s evolutionary theory tells us that the microbiome should have coevolved with us since the early days of humans. Recent studies have suggested that the hologenome, a collective genome carried by the holobiont, may be inherited between generations with reasonable fidelity. The first principles would also predict that the variations in hologenome are subject to *selection* and *drift* effects evolutionarily. Similar studies with the bacterial part of human microbiomes have suggested that both selection and drift, as well as dispersal and speciation are the four processes or mechanisms that underlie the assembly and diversity maintenance of bacterial communities (*e.g*., [6], [7], [8], [9], [10], [11], [12]). However, the question has not been addressed, to the best of our knowledge, regarding the human virome.

Human virome exists in the forms of ecological communities or assemblages of viruses [3], [4]. How ecological community is assembled (formed) and how its diversity is maintained after formation is a central topic of community ecology. In fact, Darwin wrote, in the concluding paragraph of On the Origin of Species, “It is interesting to contemplate an entangled bank, clothed with many plants of many kinds, with birds singing on the bushes, with various insects flitting about, and with worms crawling through the damp earth.” It is true that Darwin was stressing that the endless, most beautiful and wonderful forms of lives (which are essentially the ecological communities) have all evolved through the process of natural selection [13]. Nevertheless, the evolutionary theory through natural selection could not interpret how the entangled bank is formed especially on the ecological time scale. For example, Darwin’s evolutionary theory maintained that the universal struggle for life as a consequence to natural selection, then how could diverse lives (species) in entangled bank coexist. Two diametrically opposed theories explaining community assembly exist in modern community ecology to explain the mechanism of community assembly. One is classic niche theory first proposed nearly a century ago (*e.g*., [14]). Niche can be roughly defined as the sum of the habitat requirements and behaviors that allow a species to persist and produce offspring, and natural habitats can be considered as mosaics of niches suitable for different species to live and prosper. Niche theory maintains that different species occupy differentiated niches in ecological community; therefore community assembly is a deterministic process. In other words, deterministic selection forces drive the assembly of community and maintain the coexistence of many species rather than monopolized by a single species (i.e.*,* diversity maintenance).

That each species lives and prospers in its own niches also implies that niche differences influence the species abundances. In late 1990s, Stephen Hubbell challenged the niche view and he assumed that the differences among members of an ecological community of tropically similar species (*e.g*., the viral species in our gut) are neutral in the sense that the differences do not matter for their success. This assumption implies that niche differences do not influence species abundances and the abundance of each species follows a random walk—that is primarily determined by stochastic drifts of birth, death and dispersal. In other words, species are born unequal in abundances not because of their niche differences, instead, because of the randomness (stochastic drifts) in their birth/death/dispersal probabilities.

Like many scientific theories, diametrically opposed theories are rarely totally correct and a middle ground is frequently possible. In fact, several hybrid models of niche and neutral theories have been proposed (*e.g*., [9], [10], [15], [16], [17], [18], [19], [20], [21], [22], [23], [24], [25]. Therefore, ideally, studies with objectives like ours of this study should resort to niche-neutral hybrid models. However, testing niche theory with statistical rigor is already rather difficult, and so does the testing of hybrid models, primarily because the data requirements for testing niche or niche-neutral hybrid are far more demanding and especially hard to meet in the case of human microbiomes because manipulative experiments are often not allowed due to ethic constraints. Actually, even testing the neutral theory, which is much less demanding for data requirements than testing the niche or niche-neutral hybrid models, is rather challenging. This is the very reason we adopt five neutral models/approaches in this study to comprehensively cross-verify the findings regarding the test of neutral theory, as introduced in the section of Material and Methods.

The single objective of this study is to test the fitness of neutral theory, with statistical rigor, to the human virome by reanalyzing four independent datasets of human virome. The significance of the neutral-theoretic tests of the human virome answers the following fundamental question: how is the human virome assembled and how its diversity is maintained after assembling? If neutrality is prevalent in the human virome, then stochastic neutral drifts in demography (birth/death) and dispersal (migration) are primarily responsible for the patterns of observed community structures and dynamics. This is equivalently to say that deterministic selection forces play relatively small role in shaping the virome diversity patterns or driving virome dynamics. Practically, the structure and dynamics of human virome have far reaching implications to our health and diseases. For example, certain states of virome dynamics or certain patterns of viral communities might be associated with healthy hosts, and alternative states/patterns might be associated with disease. To the best of our knowledge, this study should be the first comprehensive tests of five neutral-theoretic models, including standard Hubbell’s neutral model (HNM), Sloan’s near-neutral model (SNM) and Harris et al. [26] multi-site neutral model (MSN), further augmented by Ning et al. [27] normalized stochasticity framework and Hammal et al. [28] power analysis for the neutral test (PNT), in virome ecology. The multi-model approach allows us to not only determine the relative importance of stochastic neutrality (drifts) *vs*. deterministic selection in shaping/driving viral community patterns/dynamics, but also evaluate the level of type-I and type-II errors.

## 2 Material and Methods

### 2.1 Virome datasets and bioinformatics analysis for viral OTU tables

Four published datasets of human viromes were reanalyzed in this study, with a total of 179 samples (Table S1). There were 287 samples in the four original studies and we excluded 108 diseased samples and only preserved 179 healthy samples in the present study. Since it was difficult to collect multiple datasets with the same or even very similar meta (environmental) factors, we decided simply to ignore the difference in meta factors such as host age, sex, etc. Instead, we only required that the datasets were collected for sequencing the reads of human viromes. In other words, we did not care the “heterogeneity” or lack of homogeneity among the treatments of the four datasets. Nevertheless, starting from the virome reads, we kept the exactly same computational procedures and quality control measures to get the viral OTU (operational taxonomic unit) tables. Since all of the analyses used in this study adopt treatment or group as the basic unit, *i.e*., each treatment is tested for the neutrality or non-neutrality *independently*, the potential inter-treatment (group) heterogeneity does not matter for drawing conclusions.

**Fig. 1:**
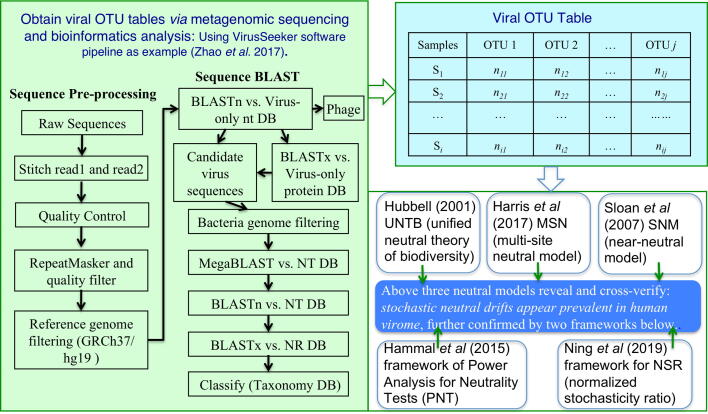
A diagram illustrating the computational pipelines and procedures for testing the neutrality of virome diversity: the left side illustrated the bioinformatics pipelines for obtaining the viral OTU table (top right); the bottom right block illustrated the five models used to test and confirm the neutrality of virome diversity. In the bioinformatics pipeline (left), sequences were clustered using CD-HIT with ≥98% identity, and we utilized BAW-MEM (v0.7.11, k = 15, L = 100,100) for mapping sequences against reference genomes.

We adopted VirusSeeker, a BLAST-based NGS data analysis software pipeline [29], to reanalyze all of the virome reads with the same configurations and to ensure consistent computational procedures were applied to obtain the viral OTU tables. Fig. 1 illustrated the flowchart of VirusSeeker, as well as the follow-up procedures for performing the power law analysis with the viral OTU tables generated from the VirusSeeker pipeline. An advantage with the VirusSeeker pipeline is that it handles both eukaryotic virus and virome composition equally well, while many other pipelines usually focus on one or the other. This advantage is rather helpful for us to obtain consistent OTU tables across the four datasets.

For Hubbell’s neutral model (HNM), each sample within each treatment is tested for neutrality.

All samples from a treatment are treated as a metacommunity and are fitted to a MSN model.

Pair-wise source–destination model for each pair of treatments is used to build a Sloan near-neutral model (SNM). For the time-series samples, only the early-late pair is allowed for building Sloan model (dataset #2).

## 3 Methods

### 3.1 Hubbell’s UNTB (unified neutral theory for biodiversity and biogeography) and Hubbell’s standard neutral model (HNM)

With the UNTB, conceptually, local communities are connected through migration to form metacommunity. It is assumed that similar neutral processes drive both the dynamics of local and metacommunities, except that in metacommunity speciation, rather than migration, is in operations [30], [31]. The *neutral process* or *ecological equivalence* between species implies that the demographic rates (birth/death) of all species are stochastic but *equivalent* on per capita basis [26]. There are three key parameters (elements) with the UNTB, the immigration probability (*m*), which controls the coupling of a local community to the metacommunity, namely the *fundamental dispersal number*. The second is the speciation rate, also known as the *fundamental biodiversity number* (*θ*), which can be interpreted as the rate at which new individuals are added to the metacommunity through speciation. The third aspect of the UNTB is to assume that the SAD (species abundance distribution) of each community sample (*X*_i_) can be described by the multinomial (MN) distribution, *i.e*.,:

(1) $$\bar{X_{i}}M\sim N\left(N_{i},\;{\bar{\pi }}_{i}\right)$$

where *N_i_* is the size of *i*-th local community, ${\bar{\pi }}_{i}$ is a vector of the probability of observing a particularly species at *i*-th local community [26], $\bar{X_{i}}$ represent vector of local community sample (*X*_i_).

With Hubbell’s UNTB or standard neutral model (HNM), Etienne’s [32], [33] exact neutrality test can be used to test the neutrality of community samples, a pseudo *P*-value is obtained to determine the difference between observed (actual) and simulated likelihoods based on the HNM. When the *P*-value >0.05, it means that both the actual likelihood and predicted likelihoods by the HNM are not distinguishable and the neutrality hypothesis (H_0_) cannot be rejected.

### 3.2 Harris et al. [26] Multi-Site neutral model (MSN)

With the previous HNM, the migration probability for each community sample is estimated independently. The advantage of this approach is its simplicity, but local communities are linked *via* migration and the migration probabilities can be different from community to community. That is, simultaneously estimating the migration rates (*I*_i_) for all local communities should be more realistic in emulating neutral theory model. In general, fitting multiple sites (local communities) UNTB with possibly different immigration rates is computationally intractable when the number of sites increases to certain level, and the computation must be approximated [26]. Harris et al. [26] approximated the neutral models with the hierarchical Dirichlet process (HDP) and developed an efficient Bayesian fitting framework to fit the multi-site neutral model (MSN), which is essentially a version of Hubbell’s UNTB allowing for potentially different migration probability (*m_i_*). Harris et al. [26] approximation encapsulated the three essential elements of Hubbell’s [30] UNTB, as mentioned previously, offering an efficient Bayesian fitting strategy for the MSN.

At the local community scale, assuming there is a potentially infinite number of species that may exist in the local community, then the stationary distribution of observing local population *i* is a Dirichlet process (DP), *i.e*.,

(2) $$\left(\bar{\pi_{i}}\right|I_{i},\bar{\beta }D\sim P\left(I_{i},\bar{\beta }\right)$$

Where $\bar{\beta }=\left(\beta_{1},...,\beta_{S}\right)$ is the relative frequency of each species in the metacommunity, and *I*_i_ is the immigration rate. $I_{i}=(m_{i}/\left(1-m_{i}\right)\left(N_{i}-1\right)$, where *m*_i_ is the immigration probability (the same as the previous HNM) to local community *i*, and *N*_i_ is the local community size.

At the metacommunity level, a Dirichlet process was still used by Harris et al. [26], the metacommunity distribution is modeled as a purely stick-breaking process, *i.e*.,

(3) $$\bar{\beta }S\sim tick\left(\theta \right)$$

where *θ* is the fundamental biodiversity number.

When both local community and metacommunity are approximated with Dirichlet processes, the problem becomes a hierarchical Dirichlet process (HDP) [26], [34]. Alternatively, Dirichlet process (DP) can also be derived from the so-called Chinese restaurant process, from which the Antoniak equation is derived.

The Antoniak equation represents the number of species (*S*) observed following *N* times of sampling from a Dirichlet process with biodiversity number *θ*:

(4) $$P\left(S\left|\theta ,N\right)\right)=s\left(N,S\right)\theta^{S}\frac{\Gamma (\theta )}{\Gamma (\theta +N)}$$

in which *s*(*N*, *S*) is the unsigned Stirling number and Γ(*.*) denotes the gamma function [54].

Coupling equations (1–4) forms the full HDP-MSN, and Harris et al. [26] developed an efficient Gibbs sampler, which is a type of Bayesian Markov Chain Monte Carlo (MCMC) algorithm, for implementing the HDP-MSN approximation. The Gibbs sample is then used to simulate neutral local- and meta-communities. Harris et al. [26] adopted a set of procedures similar to Etienne’s [32], [33] exact neutrality test, and developed the procedures to test the neutrality based on the MSN model on both local- and meta-community level, respectively. To test the neutrality at the metacommunity level, ***P_M_***, which is “the proportion of the simulated neutral samples with their likelihoods *not* exceeding (≤) the observed data likelihood” [26]. If ***P_M_*** > 0.05, the metacommunity appears to satisfy the MSN model, according to Harris et al. [26]. Similarly, there is ***P_L_***, which is the proportion of the simulated locally neutral samples not exceeding (≤) the observed data likelihood [26]. If ***P_L_*** > 0.05, the local community appears to satisfy the neutral model.

### 3.3 Sloan et al. [35], [36] near-neutral model (SNM)

Sloan et al. [35], [36] derived a near neutral model to explain the assembly mechanisms of prokaryotic communities. The model contains source and local communities, similar to “mainland” and “island” in the theory of island biogeography. A significant difference between Sloan near-neutral model (SNM) and Hubbell’s standard neutral model (HNM) is that the former does not enforce strict neutral equivalence: a species may possess competitive advantage (positively selected) or disadvantage (negatively selected). As a continuous version of Hubbell’s discrete neutral community model, Sloan’s model does not require observed species abundance distributions and can test exceptionally large prokaryotic communities. The following equations are introduced to outline the near-neutral process captured by Sloan near neutral model.

Let us assume that the local community is saturated with *N_T_* individuals. The renewal of individuals within the local commodity is as follows. One individual dies or leaves the local community and is replaced by another individual immigrating from a source community with probability *m* or by offspring of a random individual within the local community with probability (1-*m*). Then, the probability that the abundance of the *i-*th OTU increases by one individual, decreases by one individual, or unchanged can be given by:

(5) $$\mathrm{Pr}\left(N_{i}+1/N_{i}\right)=\left(1-\frac{N_{i}}{N_{T}}\right)\left[mp_{i}+\left(1-m\right)\left(\frac{N_{i}}{N_{T}-1}\right)\right]$$

(6) $$\mathrm{Pr}\left(N_{i}-1/N_{i}\right)=\frac{N_{i}}{N_{T}}\left[m\left(1-p_{i}\right)+\left(1-m\right)\left(\frac{N_{T}-N_{i}}{N_{T}-1}\right)\right]$$

(7) $$\mathrm{Pr}\left(N_{i}/N_{i}\right)=\frac{N_{i}}{N_{T}}\left[mp_{i}+\left(1-m\right)\left(\frac{N_{i}-1}{N_{T}-1}\right)\right]+\left(\frac{N_{T}-N_{i}}{N_{T}}\right)\left[m\left(1-p_{i}\right)+\left(1-m\right)\left(\frac{N_{T}-N_{i}-1}{N_{T}-1}\right)\right]$$

in which *p_i_* is the occurrence frequency of the *i-*th OTU in the source community and *N_i_* is the abundance of *i-*th OTU in the local community. Let *x_i_* = *N_i_*/*N_T_* is the occurrence frequency of the *i-*th OTU in the local community. From Sloan’s model, one can determine whether each species is neutral or not [35], [36], [37]. That is, to determine whether the observed *x_i_* of species (OTU) *i* fall within its 95% theoretical interval predicted from the neutral community model. If *x_i_* falls within the predicted interval, the species is judged to be neutral. If *x_i_* exceeded the predicted upper interval, the species is judged to be above neutral (positively selected) and the species is considered to possess a competitive advantage. Vice versa, if *x_i_* is below the predicted lower interval, the process is judged to be below neutral or negatively selected, and the species is considered to possess a competitive disadvantage.

### 3.4 Ning et al. [27] normalized stochasticity ratio (NSR)

There was concern that the UNTB might over-estimate the true strength of neutral processes, and Ning et al. [27] developed the so-termed normalized stochasticity ratio (NSR) framework as an alternative approach to gauging the “upper bounds” of the stochasticity level. The principal foundation of Ning et al. [27] mathematical framework is that deterministic processes should drive ecological communities more similar or dissimilar than null expectation of neutrality. Ning et al. [27] formulated a sophisticated procedure to implement a null model for quantifying stochasticity. A key metric in their framework was the adoption of Ružička similarity metrics, a species-abundance based similarly that generalized Jaccard binary similarity coefficient [55]. Let *C_ij_* represent the observed similarity between the *i-*th and *j-*th community,

(8) $$C_{\mathrm{ij}}=\frac{\sum_{S}\min(p_{k}^{i},p_{k}^{j})}{\sum_{S}\max(p_{k}^{i},p_{k}^{j})}$$

where *S* is the number of species, $p_{k}^{i}$ and $p_{k}^{j}$ are the relative abundance of *k-*th species in the *i-*th and *j-*th community.

Assume there exist *m* local communities in a metacommunity, *C_ij_* be the observed similarity between the *i-*th local community and the *j-*th local community in the metacommunity. Let *E_ij_* be the null expected similarity between the *i-*th community and the *j-*th community in one simulated metacommunity. Let $\bar{E_{\mathrm{ij}}}$ be the average of the null expected similarity between the *i-*th and the *j-*th communities in 1000 simulated metacommunities. Two possibilities exist in the evaluation of the community stochasticity. One is that deterministic processes drive communities more similar, in which *C_ij_* > $\bar{E_{\mathrm{ij}}}$, and the stochasticity ratio (type A *SR*) is.

(9) $$SR_{\mathrm{ij}}^{A}=\frac{\bar{E_{\mathrm{ij}}}}{C_{\mathrm{ij}}}$$

Another possibility is that deterministic processes drive communities less similar, in which *C_ij_* < $\bar{E_{\mathrm{ij}}}$, and the stochasticity ratio (type B *SR*) is.

(10) $$SR_{\mathrm{ij}}^{B}=\frac{1-\bar{E_{\mathrm{ij}}}}{1-C_{\mathrm{ij}}}$$

The stochasticity ratio in the whole metacommunity is then,

(11) $$\mathrm{SR}=\frac{\sum_{\mathrm{ij}}^{n^{A}}SR_{\mathrm{ij}}^{A}+\sum_{\mathrm{ij}}^{n^{B}}SR_{\mathrm{ij}}^{B}}{n^{A}+n^{B}}$$

in which *n^A^* represents for the number of the pair-wise similarities that are larger than null expectation, and *n^B^* represents for the number of the pair-wise similarities that are less than null expectation. *SR* measures the *strength of stochasticity* in the community assembly, and it takes the values from 0 to 100%. If the community assembly is extremely deterministic without any stochasticity, then *SR* would be 0%; otherwise *SR* would be 100%. Ning et al. [27] suggested that when expected stochasticity is very low, *SR* may overestimate stochasticity. To remedy this issue, *SR* should be normalized, and the normalized stochasticity ratio (*NSR*) exhibits higher precision than the *ST* and its exact definition and computational procedure are referred to Ning et al. [27]. We adopt the NSR in this study.

### 3.5 Checking Type-I and Type-II errors in neutrality tests and power analysis

#### 3.5.1 Type-I error, FDR (false discovery rate) control and P-threshold in neutrality tests

The previous neutrality test procedures used a significance level α = 0.05 that may lead to Type-I error, namely, incorrectly reject the true neutrality null hypothesis with a 5% probability (*i.e*., obtaining a false positive with a small probability event). When many tests are performed simultaneously in the so-termed multiple testing problem, the chance for committing Type-I error can be raised inadvertently. The false discovery rate (FDR) control is frequently used to adjust the potential bias. However, the slightly “unorthodox” convention used for testing the neutral theory made FDR adjustment inapplicable. In terms of the convention used to test neutral theory, the null hypothesis (*H*_0_) is constructed in the following manner: No significant difference exists between the actual likelihood and the theoretical likelihood predicted by the neutral theory, and whether or not an associated pseudo *P-*value computed for the likelihood difference exceeding the *P*-threshold value set for testing the null hypothesis. When a pseudo *P*-value > *P*-threshold, then the community tested is judged to satisfy the neutral model, that is, there is not significant different between the observed and neutral likelihoods. To the best of knowledge, this has been a *de facto* standard practice in virtually all tests of the neutral theory. A somewhat unexpected consequence for this standard practice is that the FDR control for correcting Type-I error is not applicable for neutrality tests. This is because FDR control can only raise the *P*-value for each test, and then can lead to higher “passing rates” (strictly speaking, “failure rates” to reject neutrality) of neutrality when the convention used in neutrality tests is adopted [11]. In other words, application of FDR may actually relax the criterion for passing neutrality tests and make the inference less strict (conservative), an obviously undesirable consequence in testing neutral theory. We believe this somewhat unorthodox convention used to test neutral theory in the existing literature, which makes FDR control impossible, is an issue that should be fixed, but rarely raised in our observation. In our opinion, it appears that there is not an easy fix to the issue unless the traditional convention is changed, but a direct change of the convention may create a dilemma in reviewing the existing literature of neutrality tests. Instead, a simple fix for dealing with the dilemma in neutrality tests can be to adopt various *P*-threshold values, as detailed in Ma [11], [12]. When the *P*-threshold is raised gradually, the bar to accept neutrality tests is raised accordingly. This fix equivalently lowers the risk of committing Type-I error without resorting to FDR.

#### 3.5.2 Type-II error and Hammal [28] framework for detecting alternative non-neutral processes

Like any statistical hypothesis tests, testing the neutral theory model also involves Type-II error—incorrectly not rejecting a false null hypothesis (*i.e*., obtaining a false negative finding). In testing neutral theory, Type-II corresponds to a popular criticism, that apparent satisfaction to the neutral theory patterns may not be attributed to true neutral processes; instead, non-neutral processes may be responsible for the similar or same patterns indistinguishable statistically from what are predicted by neutral theory model. If this objection to neutral theory is *completely* true, then neutral theory and, to a larger extent, the SAD datasets, are of little or no value in detecting the underlying mechanisms (processes) of community structures (*i.e*., community assembly and diversity maintenance). The fact of the matter seems to be, while the criticism certainly has certain merits, it cannot be true totally. One successful effort to deal with the well-known criticism is Hammal et al. [28], which purely depended on simulation efforts without resorting to wet-lab experiments that often depends on artificially controlled experiments. While the controlled wet-lab experiments can be rather involving, the simulation-based Hammal approach can be rather computationally intensive. Hammal et al. [28] developed a framework to determine *when* SAD datasets and *what* neutral models can indeed detect non-neutrality. They formulated the problem as a **power** analysis for the **neutrality test** (abbreviated as **PNT** in this article) for controlling Type-II error, and their approach is of obviously critical importance for neutral-theoretic studies like this one.

The power of a statistical test can be defined as the probability that an in-fact false null hypothesis (the alternative hypothesis is true) is correctly rejected. It is the capacity (power) to avoid type-II error, *i.e*., (1 − *β*), where *β* is Type-II error rate. Generally, three factors determine the power of a test: (*i*) the *sample size*; (*ii*) *statistical significance level* as measured by the threshold *P*-value of hypothesis testing (therefore, influenced by Type-I error; iii) the *effect size* that is quantified by the deviation from the null hypothesis. In Hammal et al. [28] framework, the effect size is controlled by the parameter value of the non-neutral models that they developed to simulate possible non-neutral processes in the SAD data to be analyzed. They introduced three non-neutral local community models and two non-neutral metacommunity models, all of which are stochastic and similar to Hubbell’s [30] standard neutral model (SNM) but driven by explicit non-neutral forces such as competitions and unequal species fitness. Hammal et al. [28] showed that the presence of non-neutral processes in SADs, which also satisfy the SNM, is detectable as long as sample size is sufficiently large and/or the effect size (amplitude of non-neutral process effect) is sufficiently strong. They concluded that, although the PNT can indeed be rather complex, computationally expensive particularly, resolving the issues related to Type-II error in analyzing SAD patterns with neutral theory models is possible. In practice, their work demonstrated that it is possible to offer convincing evidence to either support or rejected the findings from a neutral theory model, as long as sample size and/or effect size are appropriate. In the present study, we adapt their framework to check the validity of our findings from neutrality tests.

Since the power of a statistical test is dependent on which alternative hypothesis is assumed, Hammal et al. [28] framework strategically chose to demonstrate two classes of non-neutral processes: *interspecific competition* and *intrinsic* (density-independent) *fitness differences* between species. The former promotes species co-existence and the latter signals the niche differentiations. Both represent opposite ends of a spectrum of possible non-neutral processes, which could potentially be of infinite varieties. On the one end, the symmetric inter-specific competition is likely to generate equal abundances among species (hardly discernible from neutrally generated equal abundances); on the other end, the intrinsic fitness differences tend to generate heterogeneous species abundances.

Tactically, Hammal et al. [28] introduced two local-community, non-neutral, competition models: (*i*) the HL model, which is named after the authors of Haegemann & Loreau [38] that proposed a multi-species stochastic Lotka-Volterra model; (ii) the PC model, a density-dependent dynamics model similar to one studied by Pigolotti & Cencini [25]. They also introduced a third local-community non-neutral model: the intrinsic fitness (IF) model that assumes the fecundity of each species is a random variable following a Gamma distribution. Furthermore, they introduced two non-neutral metacommunity models, LOGS model described by a log-series distribution and EVEN model in which all species have equal abundances. Mathematical details of these three non-neutral local and two metacommunity models are referred to Hammal et al. [28].

When coupled with non-neutral LOGS metacommunity model, each of the three local-community non-neutral models should be equivalent to the standard HNM model when the local dynamics are actually neutral (the model control parameter is set to zero). When the dynamics becomes more non-neutral (by raising the control parameter), the deviations from the HNM (the effect size) become stronger, and then it is expected that the power of the test for the neutral null hypothesis to be lifted.

Hammal et al. [28] defined the power of the test as the proportion of non-neutral datasets (generated by one of the non-neutral local community models: HL, PC or IF) for which the test of non-neutral effect was significant. That is, the neutral null hypothesis is rejected and the alternative non-neutral process simulated by HL, PC or IF model is accepted. The small power value indicates that there is no no-neutral process or that the non-neutral process is not sufficiently strong in the metacommunity. Alternatively, the large power value indicates that there is sufficiently strong non-neutral process in the community. Finally, we compare the power test finding (PNT tests) with the finding from standard neutrality tests. If both findings are consistent, we conclude that the neutrality testing results are reasonably reliable, and the risk of incurring Type-II error is tolerable. If both findings are not consistent, we conclude that the neutrality testing results should be questioned and the risking of incurring Type-II error (obtaining false negatives) is high [11], [28].

## 4 Results

### 4.1 Hubbell’s [30] neutral model (HNM)

Hubbell’s [30] neutral model (HNM) is the first unified neutral theory model (UNTB) for biodiversity, although similar neutral theory models were already influential in molecular biology in the form of neutral theory for molecular evolution, and primitive ecological neutral model existed before Hubbell’s comprehensive UNTB was developed.

The HNM neutrality test results (Table 1, summarized from Table S2 in the online supplementary information or OSI) show that the passing rates of the human virome ranged between 55.6% and 100% with an average of 88%. The 3 treatments of dataset #3 had the lowest passing rates (55.6–84.6%), which may be to do with the sampling locations (blood and lung). All other samples except for the dataset #3 were obtained from gut and therefore the gut virome appears to have higher neutrality than the lung.

**Table 1:** The mean of Hubbell’s neutral model (HNM) parameters fitted to the human virome datasets, summarized from Table S1.

Datasets	Group	*J*	*S*	*θ*	*m*	*P-*value	Percentage (%) for passing neutrality test
Dataset #1	Urban A	24,301	176	27.248	0.996	0.950	95.0
	Village B	12,809	211	37.276	0.999	0.765	90.0
	Village C	58,423	250.5	37.621	0.994	0.998	100
	Village D	27,053	271	45.175	0.993	0.787	86.7

Dataset #2	Oct-2013	83,321	523	77.946	0.631	1.000	100
	Jan-2014	93,226	544	78.401	0.812	1.000	100
	Aug-2014	78,517	533	79.502	0.743	1.000	100

Dataset #3	Blood-Control-LTR	2324	23	5.547	0.970	0.749	84.6
	Lung-Control-LTR	3135	98	23.920	0.682	0.337	65.2
	Lung-Control-OD	2604	116	27.775	0.979	0.466	55.6

Dataset #4	Healthy	2234	50	9.437	0.982	0.924	100

Average across datasets		35,268	254	40.895	0.889	0.816	88.8 %
Standard error across datasets		10,889	59	8.089	0.043	0.069	4.6 %

*Samples with less than 100 reads were excluded from the neutrality test.

### 4.2 Ning et al. [27] normalized stochasticity ratio (NSR)

**Fig. 2:**
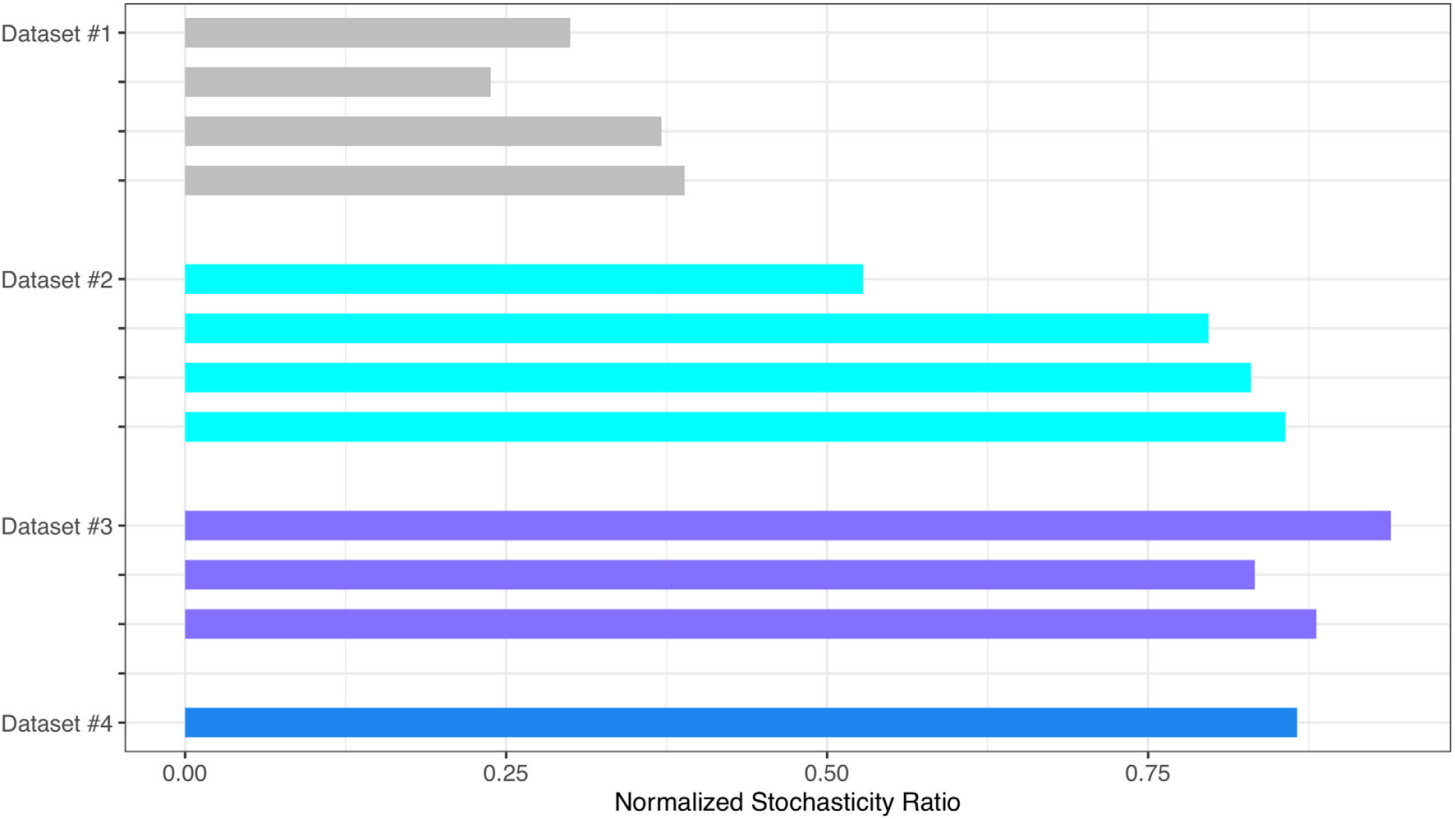
The NSR (normalized stochasticity ratio) for the 12 treatments of 4 datasets (case studies): the large NSR values indicated strong level of stochastic neutrality.

The average normalized stochasticity ratio (NSR) across the 14 treatments of the 4 datasets was 0.65 (Table 2, Fig. 2), which suggests that stochastic neutrality level should be approximately 65% on average. This confirms the finding from previous HNM tests.

**Table 2:** The mean of Ning et al. [27] similarity (S) and normalized stochasticity ratio (NSR) for each treatment of the human virome datasets.

Datasets	Treatments	*Number of Pair-wise Comparisons*	Similarity (*S*)	Normalized Stochasticity Ratio (*NSR*)
Dataset #1	Urban A	190	0.747	0.300
	Village B	45	0.725	0.238
	Village C	120	0.795	0.371
	Village D	105	0.781	0.389

Dataset #2	Oct-2013	3	0.693	0.528
	Jan-2014	28	0.848	0.797
	Apr-2014	21	0.886	0.830
	Aug-2014	66	0.907	0.857

Dataset #3	Blood-Control-LTR	120	0.878	0.939
	Lung-Control-LTR	630	0.919	0.833
	Lung-Control-OD	465	0.931	0.881

Dataset #4	Healthy	10	0.956	0.866

**Mean**		150	0.839	0.652
**Standard Error**			0.025	0.076

### 4.3 Sloan’s [36] neutral model (SNM)

**Fig. 3:**
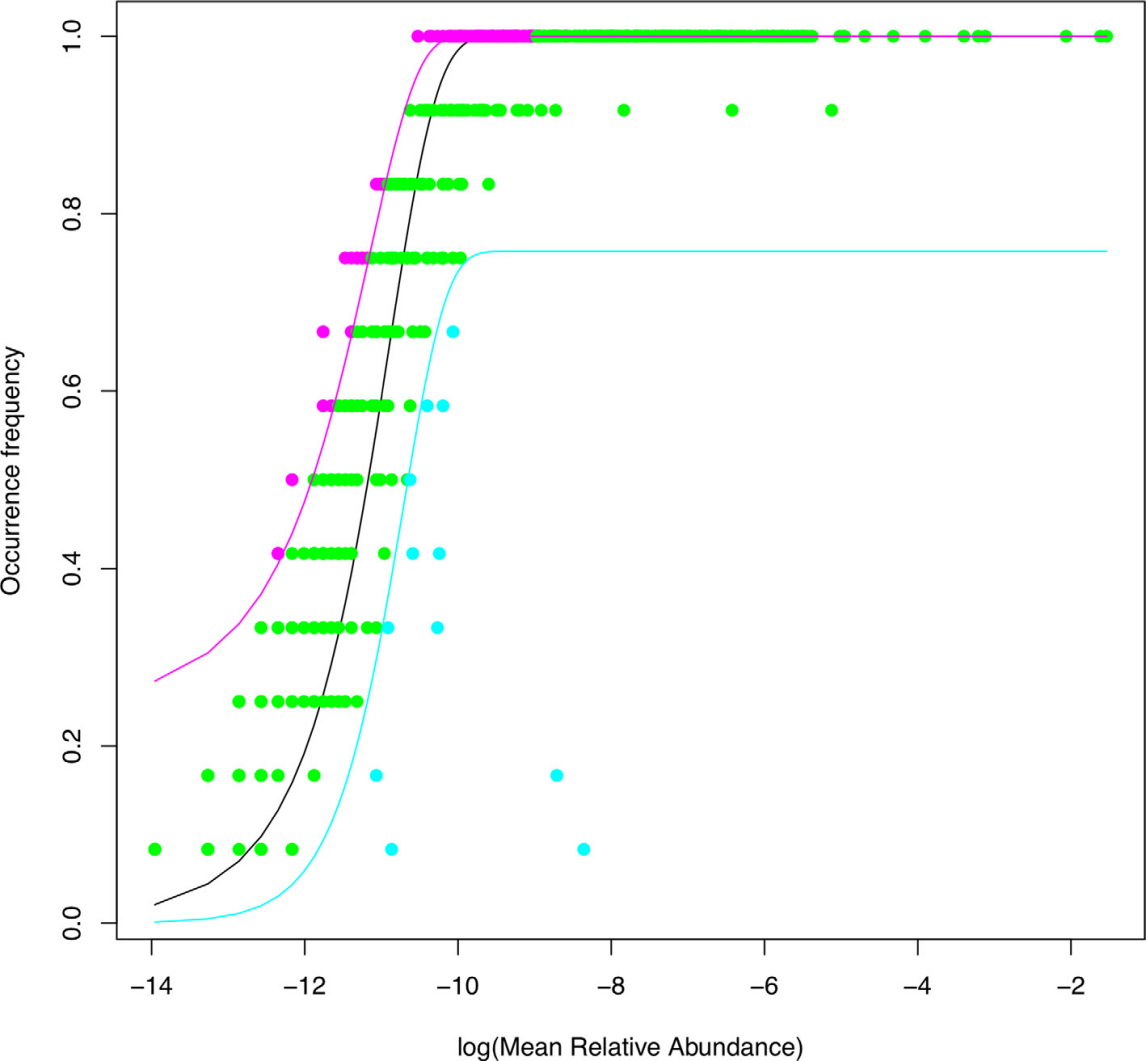
The Sloan [35], [36] near neutral model fitted to the “Aug-2014” treatment of Dataset #2, suggesting that most species are neutral (green dots), and small numbers are positively selected (pink dots) or negatively selected (cyan dots). (For interpretation of the references to colour in this figure legend, the reader is referred to the web version of this article.)

The results from fitting Sloan neutral model to the human virome datasets suggest that the abundances of approximately 67% species are consistent with the prediction of the neutral model (Table 3, Fig. 3). The average immigration rate (probability) (*m*) or fundamental dispersal number ranged from 0.336. The percentage of species whose abundances are higher than predicted by Sloan model is approximately 22%, which is termed as positively selected species. The percentage of species whose abundances are lower than predicted by Sloan model is approximately 10%, which is termed as positively selected species. Therefore, we conclude that at species level, stochastic neutral forces (neutral drifts in demography and dispersal) are likely to be responsible for determining the species abundances of approximately 67% species. That is stochastic neutrality play a major role given its influences reach 2/3 viral species.

**Table 3:** Fitting of the human virome datasets to Sloan’s [35], [36] near neutral model*.

Datasets	Source Community	Destination Community	*N*	Immigration Probability (*m*)	*R*^2^	Total Number of Species	Percentage of Species Below Neutral (%)	Percentage of Neutral Species (%)	Percentage of Species Above Neutral (%)
Dataset #1	Village B	Urban A	24,301	0.046	0.265	478	14.4	65.1	20.5
	Village B	Village C	58,423	0.174	0.128	511	11.2	66.9	21.9
	Village B	Village D	27,053	0.262	0.094	505	12.3	64.4	23.4
	Village C	Urban A	24,301	0.014	0.031	541	15.0	60.6	24.4
	Village C	Village D	27,053	0.089	0.172	594	14.6	67.2	18.2
	Village D	Urban A	24,301	0.024	0.110	551	14.0	69.7	16.3
	Village D	Village B	12,809	0.052	0.093	505	10.9	80.4	8.7
	Village D	Village C	58,423	0.072	0.189	594	12.5	70.0	17.5

Dataset #2	Oct-2013	Jan-2014	93,226	1.127	0.109	594	2.7	67.7	29.6
	Oct-2013	Aug-2014	96,039	1.135	0.349	597	3.2	64.3	32.5
	Jan-2014	Aug-2014	96,039	0.929	0.661	674	3.7	67.8	28.5
	Apr-2014	Aug-2014	96,039	0.108	0.772	686	4.5	65.0	30.5

**Mean**			53,167	0.336	0.248	569	9.9%	67.4%	22.7%
**Standard Error**			9795	0.129	0.068	19	1.416	1.397	2.006

* Some samples that failed to fit Sloan near neutral model (negative *R*^2^) are not list here.

In above interpreted results from Sloan near-neutral modeling (Table 3, Fig. 3), the source and destination communities were set to different sets of virome samples, which is *de facto* standard scheme for applying Sloan model. Alternatively, both source and destination communities could be set to the same sets of virome samples; Table S5 in the OSI exhibited such results from Sloan modeling. With the alternative scheme, the neutrality level or the proportions of neutral species increased to 80%, compared with 67% in the previous paragraph where source and destination communities are set differently. The proportion of positively selected species decreased to 14.4% from 22%. These comparative results should be expected, as determined by the difference in their test schemes. When the source and destination are set to same virome samples, both the similarity and corresponding neutrality should rise. This higher neutrality of 80% is closer to the neutrality level discovered with Hubbell’s standard neutral model, interpreted previously.

### 4.4 Harris et al. [26] Multisite neutral model (MSN)

Table 4 shows the results of fitting Harris et al. [26] HDP-MSN model to the human virome datasets, and the percentage for passing the MSN neutrality tests is 91.7%. What the MSN describe is actually the landscape scale because the virome of each individual is a metacommunity of viruses, and community of metacommunity is equivalent to landscape.

**Table 4:** Fitting the HDP-MSN model to the human virome datasets (with all samples from a single treatment are treated as a metacommunity described by a HDP-MSN model.

Dataset	Treatment	*θ*	*M-value*	Metacommunity			Local Community		
				*N_M_*	*N*	*P_M_*	*N_L_*	*N*	*P_L_*
Dataset #1	Urban A	176.930	53.841	2500	2500	1.000	2500	2500	1.000
	Village B	152.340	125.902	2500	2500	1.000	2500	2500	1.000
	Village C	171.999	103.245	2500	2500	1.000	2500	2500	1.000
	Village D	164.331	162.840	2500	2500	1.000	2500	2500	1.000

Dataset #2	Oct-2013	150.349	532.679	1924	2500	0.770	2500	2500	1.000
	Jan-2014	135.137	573.799	1178	2500	0.471	2500	2500	1.000
	Apr-2014	138.761	561.640	105	2500	0.042	2500	2500	1.000
	Aug-2014	126.480	582.104	386	2500	0.154	2500	2500	1.000

Dataset #3	Blood-Control-LTR	112.662	6.885	2462	2500	0.985	2437	2500	0.975
	Lung-Control-LTR	102.194	119.136	2496	2498	0.999	1487	2498	0.595
	Lung-Control-OD	113.817	106.854	2499	2499	1.000	1425	2499	0.570

Dataset #4	Healthy	96.124	22.823	2457	2500	0.983	2124	2500	0.850

**Mean**		136.760	245.979	1958.9	2499.8	0.784	2289.4	2499.8	0.916
**Standard Error**		7.838	68.717	257.8	0.2	0.103	116.6	0.2	0.047
**Passing Percentage (%)**						91.7			100

**Fig. 4:**
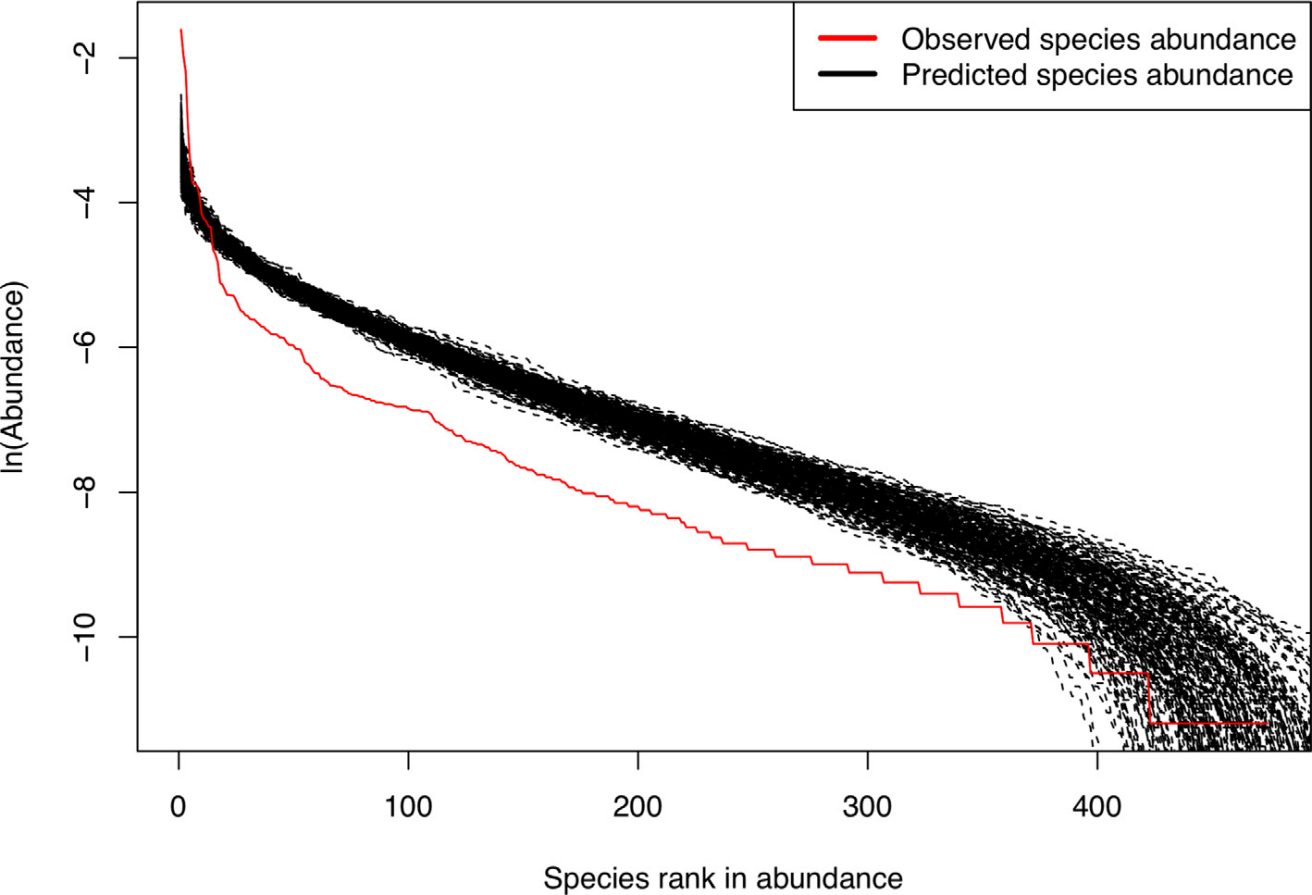
Fitting Harris et al. [26] MSN (multi-site neutral model) with “Lung-Control-LTR” treatment dataset.

The results here from the MSN modeling are fully consistent with previous test results based on Hubbell’s [30] standard neutral model, Ning et al. [27] normalized stochasticity ratio (NSR) and Sloan [35], [36] near-neutral model. These findings lead to a consistent conclusion, that is, the stochastic neutral drifts play a dominant role in virome assembly at species (Sloan), community/metacommunity (Hubbell, NSR), and landscape (MSN) levels. At all three levels, the neutrality levels exceed 50% (65–92%), as suggested by the NSR (65%) (Table 2) and by the MSN (92%) (Table 4, Fig. 4). The final sub-section of this results section address a possible challenge to the conclusion of neutrality dominance. In the following, the power analysis for the neutrality test is used to test the robustness of neutrality test by assessing the level of alternative non-neutral processes as interpreted below.

### 4.5 Power analysis for the neutrality test (PNT)

The final part of our results exposition is to interpret the PNT (power analysis for the neutral test) based on Hammal et al. [28] for detecting possible existence of non-neutral processes. The power analysis is designed to address the issue of Type-II error, *i.e*., incorrectly failing to reject a false null hypothesis (*i.e*., obtaining a false negative finding). This corresponds to a common criticism that argues for the possibility of apparent satisfaction to the neutral theory patterns could not be due to the neutral processes, instead, due to non-neutral processes that may generate the similar or same patterns indistinguishable statistically from what are predicted by neutral theory model.

Table S3 listed the parameters from both neutral (based on Hubbell’s UNTB) and non-neutral (*i.e*., power analysis based on IF and PC models) tests, including the *J, S, θ, m* and *P*-value from the neutral test with Etienne’s sampling formula (i.e.*,* Hubbell’s UNTB); *Ave. P-*value (IF) model and *Ave. P-*value (PC) (the average of *P*-values from non-neutral datasets generated by IF and PC non-neutral models respectively), as well as corresponding *Power* values. Table S4 is a summary version of Table S3, which exhibited average parameters from Table S3.

In Table 5, which is further summarized from Table S4, the first result column of *P*-values (after the three columns describing dataset information) is the *P*-value from standard neutrality test based on Hubbell’s neutral model (HNM). This column is the same as that of Table 1 for testing the HNM. *P*-value > 0.05 suggests that the community is indistinguishable from neutral. The next four columns of Table 5 presented the essential statistics of the power analysis, based on which we can draw the following findings and/or conclusions.

**Table 5:** The results from PNT (power analysis for the neutrality tests) excerpted from Table S3.

Dataset	Treatment	Parameter	Neutrality test	PNT: *P*-value from non-neutrality tests (with non-neutral datasets from simulation)			
			*P-value from Neutrality Test*	*P-value from* IF model	*P-value from* PC model	*Power of* IF model	*Power of* PC model
Dataset #1	Urban A	Mean	0.950	0.509	0.509	0.008	0.008
		Standard Error	0.049	0.007	0.007	0.005	0.005
	Village B	Mean	0.765	0.516	0.516	0.016	0.016
		Standard Error	0.116	0.014	0.014	0.007	0.007
	Village C	Mean	0.997	0.527	0.527	0.005	0.005
		Standard Error	0.003	0.009	0.009	0.003	0.003
	Village D	Mean	0.772	0.529	0.529	0.007	0.007
		Standard Error	0.097	0.012	0.012	0.003	0.003

Dataset #2	Blood-Control-LTR	Mean	0.749	0.456	0.456	0.009	0.009
		Standard Error	0.094	0.010	0.010	0.007	0.007
	Lung-Control-LTR	Mean	0.337	0.462	0.410	0.050	0.101
		Standard Error	0.079	0.030	0.037	0.016	0.030
	Lung-Control-OD	Mean	0.466	0.514	0.518	0.029	0.024
		Standard Error	0.104	0.016	0.017	0.006	0.005

Dataset #3	Healthy	Mean	0.924	0.454	0.454	0.020	0.004
		Standard Error	0.034	0.020	0.014	0.011	0.004

Percentage with *Non-neutrality* detected (%) with IF or PC non-neutral model				25.9% (30/116)	24.1% (28/116)		

Percentage with *Non-neutrality* detected but passed neutrality test (%) (False positive cases)				3.4 % (4/116)	1.7% (2/116)		

The column of *P*-value-IF > 0.05 but <*P*-value from neutrality test indicates that the non-neutral process represented by IF model is not strong enough to revoke the neutral test conclusion. Similarly, the column of *P*-value-PC > 0.05 but <*P*-value from neutrality test indicates that non-neutral process represented by PC model is not strong enough to revoke the neutral test conclusion. The small Power-value of IF (or PC) model (i.e.*,* the last two columns in Table 5) indicates weaker non-neutral process represented by IF (or PC) model.

If *P*-value > Ave.*P-*value of *IF or PC* non-neutral model, we can conclude that there is no non-neutral process or the non-neutral process is not strong enough to explain the neutrality represented by the *P*-value. Otherwise, we have detected the non-neutral process. Acceding to the IF non-neutral model, in approximately 26% (30/116) of viral communities (samples) (highlighted in grey or red in Table S3, and summarized in Table 5), the non-neutral process is detected. The percentage is slightly low with PC model, and is equal to 24% (28/116). These percentages measure the non-neutrality level in the virome, which are approximately 1/3. This number is also consistent with the percentage of neutrality measured by the NSR (65%, Table 2); theoretically and ideally the non-neutrality + NSR should be 100% (≈1/3 + 65%).

**Fig. 5:**
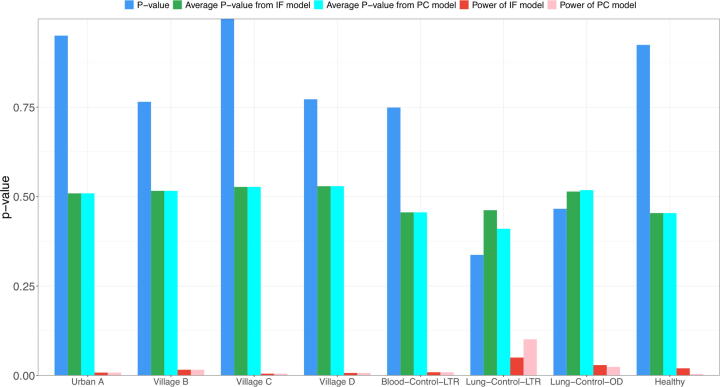
The power analysis for the neutrality tests with Hubbell’s UNTB (plotted based on Table 5): The small power values (Power of IF model and PC model) demonstrate that there is no no-neutral process or that the non-neutral process is not sufficiently strong in the metacommunity of human virome, and therefore, the findings based on the neutrality tests are reasonably robust.

The focus of power analysis is to detect the cases when *P*-value < Ave.*P-*value and *P*-value > 0.05. In these cases, although we cannot reject the neutrality hypothesis, we have detected the non-neutral process in the community that may be confounding the supposed neutral effects suggested by the *P*-value. These are false negative cases from neutrality test perspective (Hubbell’s neutral model in this article)—incorrectly failing to reject a false null hypothesis (*i.e*., obtaining a false negative finding). As revealed in Table S3 (highlighted in red), the number of false negative cases was 4 in terms of the IF non-neutral model or 2 in PC model (Table 5, Fig. 5), which are included in the 30 (IF model) or 28 (PC model) total cases of non-neutral processes detected by the power analysis as mentioned previously. The 4 (or 2) out of 116 cases indicate that in approximately 3% (2%) cases, the non-neutral processes can contribute confounding effects on the neutrality test, which is a rather low percentage and suggests that the power of neutrality test is exceptionally high. Therefore, the power analysis in this sub-section confirms the reliability (robustness) of the neutrality test from Hubbell’s neutral model.

## 5 Conclusions and discussion

Disentangling the mechanisms underlying entangled banks or revealing the mechanisms shaping the viral community assembly and diversity maintenance is rather challenging. First, manipulative experiments that could be designed to reconstruct the community assembly processes are usually infeasible, especially in studies of human microbiome and/or virome. For this reason, ideal data, particularly quantitative datasets, for detangle the mechanisms are hardly available. Second, for the previous reason, species abundance distributions (SAD) in the form of OTU tables are often the only available datasets for mechanistic analyses. This is indeed true, but there have been many critics on the usage of SAD data for testing the neutral theory. Two major critics for using the SAD datasets to testing the neutral theory for the purpose to investigating the underlying mechanisms for community assembly and diversity maintenance are: (*i*) the neutral models overestimate the neutral effects, (*ii*) the observed SAD patterns that satisfy the predictions of neutral theory models may actually be generated by non-neutral processes. In other words, both neutral and non-neutral processes (forces) may produce indistinguishable SAD patterns. Besides using Hubbell’s standard neutral model (HNM) as basic model for testing the neutral theory, we use Ning et al. [27] NSR (normalized stochasticity ratio) to gauge the minimal level of stochasticity (neutrality) level, which address the critic (*i*). To address critic (*ii*), we applied Hammal et al. [28] power estimation for the neutral test (PNT) to detect the possible existence of non-neutral processes. Aided by Ning et al. [27] and Hammal et al. [28] approaches, we not only provided reasonably strong cross-verification for the test results revealed by Hubbell’s HNM model.

While the three models of Hubbell’s HNM, Ning et al. [27] and Hammal et al. [28] offered comprehensive and robust tests of the neutral theory at the community/metacommunity levels, we further used Harris et al. [26] multi-site neutral model to explore the virome neutrality at landscape scale, and Sloan [35], [36] near-neutral model (SNM) to identify the neutral, positively selected and negatively selected species (i.e.*,* at the species level). Therefore, our study offers comprehensive testing of the stochastic neutral forces in driving virome assembly across virtually all ecological scales from population, community, and metacommunity to landscape. These comprehensive analyses concluded that the neutrality level (or passing rates of neutrality tests) ranged from 88.8% (single site neutral model) to 91.7% (multi-site neutral model) at the community/metacommunity/landscape scale. At species level, the neutral species ranged from 67.4% to 80.0%, positively selected species ranged from 14.4% to 22.7%, and negatively selected species ranged from 5.6% to 9.9%. Ning et al. [27] suggested that the lower bound of neutrality should be 65% (0.652 on the scale between 0 and 1). Finally, Hammal et al. [28] power analysis suggests that the non-neutrality is 26–28%, and among which only 2–3% may have exerted confounding effects on the neutrality test based on Hubbell’s standard neutral model.

In summary, all of the results from the five neutral-theoretic models (approaches) (*i.e*., Hubbell’s neutral model, Sloan’s near neutral model, Harris MSN, Ning NSR, and Hammal power analysis) point to one conclusion: the stochastic neutral drifts seem to be prevalent in driving the virome assembly and its diversity maintenance across scales from viral population to landscape. Roughly, the neutrality level exceeds at least ½ and is approximately 2/3, while the non-neutrality level is approximately 1/3. The false negative rate is approximately 2–3%. Given these findings, we further postulate that deterministic forces may play a relative low role in shaping/driving viral community patterns/dynamics.

Although the neutral theory of *molecular evolution* for viruses has been extensively investigated since the 1990s (e.g.*,* [39], [40], to the best of our knowledge, the neutral theory of biodiversity for virome or viruses has not been addressed previously. Arguably, the only exception is the work by Anthony et al. (2015) who wrote “we clarify that it is not our intention at this time to determine the process behind non-randomness, as these might involve a variety of either neutral processes assuming ecological equivalence or processes based on ecological niche differentiation” [56]. In that study, Anthony et al. (2015) sampled macaque feces across nine sites in Bangladesh and used consensus PCR and sequencing to identify 184 viruses from 14 viral families [56]. They used network modeling and statistical null-hypothesis testing to detect the existence of non-random deterministic patterns between the nine sites and within individual macaques. They concluded that determinism is an important process in virome assembly but is not absolute. Compared with their study with primates (Rhesus macaques), our study reveals a seemingly more prevalent randomness or neutrality in the human virome than in the primates.

Finally, we should note possible limitations of this study. Although we have utilized virtually all major neutral-theoretic models, the virome datasets we were able to collect are of limitations. This limitation is not specific to our study; instead, it has to with the state-of-the-art technology in virus identifications. Our body is inhabited by both prokaryotic (mostly bacterial) viruses and eukaryotic (mostly human) viruses. In early days, much of the efforts have been focused on eukaryotic viruses (such as influenza, HIV and Ebola) thanks to their conspicuous impacts on human health. Realizing that prokaryotic viruses can significantly affect human health by influencing the structure and function of the bacterial communities that symbiotically interact with human hosts is a recent advance. The bacteriophages, or the viruses that infect bacteria, have been found to play a critical role in shaping the bacterial community structure and function. In the case of human gut virome, as in other environments, bacteriophages dominate over other viruses in the gut ecosystem [41]. Indeed, bacteriophages are the most abundant group of viruses and are obligatorily parasites propagating in bacterial hosts, and human gut virome consists mostly of bacteriophages [41]. For this reason, approximately 78% of sequencing reads and 86% viral OTUs in the datasets we used in this study are actually bacteriophages. Therefore, the results we obtained in this study should just reflect this predominance of bacteriophages. It will be interesting to compare our findings with the results from other virome datasets in which the proportion of bacteriophages differ significantly from what we used.

Large-scale studies of microbiome are mostly started with the human microbiome project (HMP), and have been going on for slightly more than a decade. The study of virome is further behind that of bacterial microbiome [3], [4], [12]. In both cases, the late start was primarily due to our incapacity to readily culture or detect them. The difficulty is particularly serious in virome research. This is because there is not yet a universal 16S ribosomal RNA equivalent, as in bacteria, allowing for rapid taxonomic characterization of viruses. For this, metagenomic sequencing of all DNA or RNA in a sample (human, bacterial, and viral), and then computationally aligning the massive number of sequences to identify those that resemble known viral genes, have been the primary technology [29], [42]. The whole-genome sequencing is not only costly, but also computationally time-consuming. An improvement for this approach resorts to filtering samples to purge eukaryotic cells and bacteria so that only virus-like particles (VLPs) are sequenced. This technique significantly lowers the sequencing cost and reduces the computational time. Nevertheless, since the virome consists of both temperate bacteriophages within bacterial genomes and free VLPs, both total and VLP sequencing should provide greater representation of all viruses [43].

Besides the previously discussed difficulties associated with sequencing virome, many of the viral reads cannot be aligned to virome species in existing bioinformatics databases such as NCBI databases, due to our limited identification knowledge of virus species [12], [29], [42]. Although *de novo* assembly has been widely used in virology research, the effectiveness of de novo assembly for large-scale virome studies may be limited due to the complexity of viral metagenomes and the excessive micro-diversity of phages [44], [45]. In addition, using *de novo* assembly for large virome study can also be computationally expensive. Yet, another serious computational challenge that is specific to the neutral theoretic studies for virome is that the virome reads for some viral species are particularly large. When the number of reads exceeds 30,000, most existing software packages for neutrality tests could be overwhelmed because extensive simulations needed to simulate the demography and dispersal of (*e.g*., those 30,000) individuals. The neutral theory was developed in community ecology of plants and animals, in which 30,000 individuals, is not a small number at all. Therefore, it seems that there is not an easy solution for this problem either since the simulations of large number of individuals in either microbial or macrobial communities are not an easy computational task at all.

Many alternative packages to VirusSeeker [29] that is used in this study are available (see detailed reviews by Liang and Bushman [4], Sommers et al., 2021 [57]). For example, Lin et al. [46] web pipeline (VIPIE) can process multiple NGS samples in parallel, and Vilsker et al. [47] Genome Detective (GD) software package is designed for virus identification from high-throughput sequencing data. We used more recent GD software [47] to reanalyze one of the virome datasets and listed the comparative results of GD and VirusSeeker in Table S6. The VirusSeeker appears to be more powerful in identifying virus species (OTUs), but GD appears more capable in identifying virome reads. Note that this limited comparison obviously should not be used to evaluate the performance or merits of both the packages, which requires far more comprehensive evaluations in future. Both GD (https://www.genomedetective.com/) and VIPIE have been in active updates since their initial releases and both can identify SARS-CoV-2 (COVID-19 virus) efficiently.

Traditionally, most ecological theories have been developed and tested in macrobial ecology of plants and animals. The human microbiome project (HMP) triggered the avalanches of the expansions and tests of classic ecological theories in microbial ecology, thanks to revolutionary metagenomic sequencing technology, which made the generation of the microbial species abundance distribution (SAD) datasets even more accessible than that of macrobial SADs. Nevertheless, two issues arise from this gold rush in microbial ecology. First, the validity of classic ecological theories originated in macrobial ecology in microbial ecology is not automatic, and instead, microbes may possess some unique characteristics possibly different from plants and animals. Second, most microbial ecology studies have been performed with bacteriomes, rather than with viromes for the reasons discussed previously, *i.e*., lacking a universal virus marker gene similar with 16S-rRNA for bacteria and difficulties in aligning viral sequencing reads to existing virome databases. This made virome ecology lags behind both microbial ecology and macrobial ecology significantly. For example, Sommers et al. (2021) called for integrating viral metagenomics into an ecological framework and presented a comprehensive review on existing literatures on the relevant topics [57]. Ecological dimension (framework) is largely missing in traditional virology research, and virome ecology should be established to provide frameworks for investigating populations of diverse virus variants, communities of interacting viruses, virome ecosystems and landscapes [57]. For another example, Liang & Bushman’s [4] review highlighted a few consensuses and mainstream hypotheses: (*i*) Most viral reads from typical metagenomic sequencing studies are still unidentified with existing virome databases. (*ii*) There may be disease/health-specific viral community states, a postulation borrowed from studies on bacteriomes. (*iii*) Emphasized the critical significance of studies on the assembly, composition and dynamics of the human virome as well as host–virome interactions in health and disease. Besides classic ecological theories or theoretical ecology, computational biology and bioinformatics are essential for virome ecology (e.g.*,* [45]).

In spite of the previously mentioned difficulties in studies of virome ecology, advances have been made steadily in recent years. Below are some specific examples reported in last couple of years. Cebriá-Mendoza et al. [48] demonstrated that even healthy blood contains both bacteria and viruses, somewhat contrary to knowledge in traditional textbooks of medicine. Gregory et al. [49] revealed that the diversity patterns of gut viromes are age-dependent. Zuo et al. (2020) suggested that human gut-DNA virome is more heterogeneous than bacteriome in several Chinese cohorts, but they did not give precise description for how heterogeneity was measured [58]. Their primary finding that the gut virome diversity and composition are influenced by geography, ethnicity and urbanization, is similar to the case of the bacteriomes [58].

The previously mentioned examples of virome ecology research focused on the baseline virome in healthy human cohorts. Equally, if not more, important studies on virome-associated diseases have been conducted. Indeed, virome ecology is also critical for investigating virome-associated diseases. Cao et al. [50] found that the integrated gut virome and bacteriome dynamics are associated with severity of COVID-19 infections. Iorio et al. [51] reviewed the virome-bacteriome cross-correlation with the host-metabolome, which consequently influences the progression and severity of respiratory infections such as COVID-19. Li et al. [52] reviewed the interactions between virome and host immune system: how gut viruses are sensed by immune system, and in turn, modulate host immune responses during homeostasis and disease.

Bacteriophages are natural predators of bacteria since they can precisely edit the bacterial microbiota. In the case of pathogenic bacteria, studies of their phages can open potential novel treatment to fatty liver diseases and cirrhosis [53]. Szafrański (2021) suggested that oral bacteriophage is of potential therapeutic utility for killing periodontopathic bacteria, which frequently forms biofilms resistant to many antibiotics [59].

## Declaration of Competing Interest

The authors declare that they have no known competing financial interests or personal relationships that could have appeared to influence the work reported in this paper.

## References

[1] Sharon R.EG., Zilber-Rosenberg I. (2009). The hologenome theory of evolution contains Lamarckian aspects within a Darwinian framework. Environ Microbiol.

[2] Rosenberg E., Zilber-Rosenberg I. (2018). The hologenome concept of evolution after 10 years. Microbiome.

[3] Kumata R., Ito J., Takahashi K. (2020). A tissue level atlas of the healthy human virome. BMC Biol.

[4] Liang G., Bushman F.D. (2021). The human virome: assembly, composition and host interactions. Nat Rev Microbiol.

[5] Carding S.R., Davis N., Hoyles L. (2017). Review article: the human intestinal virome in health and disease. Aliment Pharmacol Ther.

[6] Li L.W., Ma Z.S. (2016). Testing the neutral theory of biodiversity with human microbiome datasets. Sci Rep.

[7] Li L.W., Ma Z.S. (2020). Species sorting and neutral theory analyses reveal archaeal and bacterial communities are assembled differently in hot springs. Front Bioeng Biotechnol.

[8] Li W., Ma Z.S. (2020). A theoretic approach to the mode of gut microbiome translocation in SIV-infected Asian macaques. FEMS Microbiol Ecol.

[9] Ma ZS. Critical network structures and medical ecology mechanisms underlying human microbiome-associated diseases. *iScience*, 2020;23(6):101195. https://doi.org/10.1016/j.isci.2020.101195. 10.1016/j.isci.2020.101195 PMC7303986 32559728

[10] Ma Z.S. (2020). Niche-neutral theoretic approach to mechanisms underlying the biodiversity and biogeography of human microbiomes. Evol Appl.

[11] Ma Z.S. (2021). Cross-scale analyses of animal and human gut microbiome assemblies from metacommunity to global landscape. mSystems.

[12] Ma Z.S. (2021). Spatial heterogeneity analysis of the human virome with Taylor’s power law. Comput Struct Biotechnol J.

[13] Nature Editorials. The entangled bank unravels. Vol. 462 | Issue no. 7271 | 19 November 2009. 10.1038/462251a 19924163

[14] Grinnell J. (1917). The niche-relationships of the California Thrasher. Auk.

[15] Fisher C.K., Mehta P. (2014). The transition between the niche and neutral regimes in ecology. Proc Natl Acad Sci USA.

[16] Stokes C.J., Archer S.R. (2010). Niche differentiation and neutral theory: an integrated perspective on shrub assemblages in a parkland savanna. Ecology.

[17] Tilman D. (2004). Niche tradeoffs, neutrality, and community structure: A stochastic theory of resource competition, invasion, and community assembly. Proc Natl Acad Sci USA.

[18] Tang J., Zhou S. (2013). Hybrid niche-neutral models outperform an otherwise equivalent neutral model for fitting coral reef data. J Theor Biol.

[19] Jeraldo P., Sipos M., Chia N. (2012). Quantification of the relative roles of niche and neutral processes in structuring gastrointestinal microbiomes. Proc Natl Acad Sci USA.

[20] Kalyuzhny M., Seri E., Chocron R. (2014). Niche versus neutrality: a dynamical analysis. Am Nat.

[21] Kalyuzhny M., Kadmon R., Shnerb N.M. (2014). A generalized neutral theory explains static and dynamic properties of biotic communities. Quant Biol.

[22] Kalyuzhny M., Kadmon R., Shnerb N.M. (2015). A neutral theory with environmental stochasticity explains static and dynamic properties of ecological communities. Ecol Lett.

[23] Noble A.E., Fagan W.F. (2015). A niche remedy for the dynamical problems of neutral theory. Theor Ecol.

[24] Ofiteru I.D., Lunn M., Curtis T.P. (2010). Combined niche and neutral effects in a microbial wastewater treatment community. Proc Natl Acad Sci USA.

[25] Pigolotti S., Cencini M. (2013). Species abundances and lifetimes: From neutral to niche-stabilized communities. J Theor Biol.

[26] Harris K., Parsons T.L., Ijaz U.Z. (2017). Linking statistical and ecological theory: Hubbell's Unified Neutral Theory of Biodiversity as a Hierarchical Dirichlet Process. Proc IEEE.

[27] Ning D., Deng Y., Tiedje J.M., Zhou J. (2019). A general framework for quantitatively assessing ecological stochasticity. Proc Natl Acad Sci USA.

[28] Hammal O.A., Alonso D., Etienne R.S. (2015). When Can Species Abundance Data Reveal Non-neutrality?. PLoS Comput Biol.

[29] Zhao G., Wu G., Lim E.S. (2017). VirusSeeker, a computational pipeline for virus discovery and virome composition analysis. Virology.

[30] Hubbell SP. The unified neutral theory of biodiversity and biogeography. *Princeton University Press*, 2001. 10.1016/j.tree.2011.03.024 21561679

[31] Hubbell S.P. (2006). Neutral theory and the evolution of ecological equivalence. Ecology.

[32] Etienne R.S. (2005). A new sampling formula for neutral biodiversity: A new sampling formula. Ecol Lett.

[33] Etienne R.S. (2007). A neutral sampling formula for multiple samples and an ‘exact’ test of neutrality. Ecol Lett.

[34] Teh Y.W., Jordan M.I., Beal M.J., Blei D.M. (2006). Hierarchical Dirichlet processes. J Am Stat Assoc.

[35] Sloan W.T., Lunn M., Woodcock S., Head I.M., Nee S., Curtis T.P. (2006). Quantifying the roles of immigration and chance in shaping prokaryote community structure. Environ Microbiol.

[36] Sloan W.T., Woodcock S., Lunn M., Head I.M., Curtis T.P. (2007). Modeling taxa-abundance distributions in microbial communities using environmental sequence data. Microb Ecol.

[37] Burns A.R., Stephens W.Z., Stagaman K. (2016). Contribution of neutral processes to microbial community assembly over host development. ISME J.

[38] Haegeman B, Loreau M. A mathematical synthesis of niche and neutral theories in community ecology. *J Theor Biol* 2011;169: 150–65. 10.1016/j.jtbi.2010.10.006 20946903

[39] Gojobori T., Moriyama E.N., Kimura M. (1990). Molecular clock of viral evolution, and the neutral theory. Proc Natl Acad Sci USA.

[40] Duchene S., Featherstone L., Haritopoulou-Sinanidou M. (2020). Temporal signal and the phylodynamic threshold of SARS-CoV-2. Virus Evol.

[41] Garmaeva S., Sinha T. (2019). Studying the gut virome in the metagenomic era: challenges and perspectives. BMC Biol.

[42] Callanan J., Stockdale S.R., Shkoporov A. (2021). Biases in viral metagenomics-based detection, cataloguing and quantification of bacteriophage genomes in human faeces, a review. Microorganisms.

[43] Adiliaghdam F., Jeffrey K.L. (2020). Illuminating the human virome in health and disease. Genome Med.

[44] Sutton T.D.S., Clooney A.G., Ryan F.J. (2019). Choice of assembly software has a critical impact on virome characterization. Microbiome.

[45] Mirzaei K.M., Xue J., Costa R. (2021). Challenges of studying the human virome—relevant emerging technologies. Trends Microbiol.

[46] Lin J., Kramna L., Autio R., Hyöty H., Nykter M., Cinek O. (2017). VIPIE: web pipeline for parallel characterization of viral populations from multiple NGS samples. BMC Genomics.

[47] Vilsker M., Moosa Y., Nooij S., Fonseca V., Ghysens Y., Dumon K. (2019). Genome Detective: an automated system for virus identification from high-throughput sequencing data. Bioinformatics.

[48] Cebriá-Mendoza M., Bracho M.A., Arbona C., Larrea L., Díaz W., Sanjuán R. (2021). Exploring the diversity of the human blood virome. Viruses.

[49] Gregory A.C., Zablocki O., Zayed A.A., Howell A., Bolduc B., Sullivan M.B. (2020). The gut virome database reveals age-dependent patterns of virome diversity in the human gut. Cell Host Microbe.

[50] Cao J., Wang C., Zhang Y., Lei G., Xu K., Zhao N. (2021). Integrated gut virome and bacteriome dynamics in COVID-19 patients. Gut Microbes.

[51] Iorio A., Biazzo M., Gardini S., Muda A.O., Perno C.F., Dallapiccola B. (2022). Cross-correlation of virome-bacteriome-host-metabolome to study respiratory health. Trends Microbiol.

[52] Li Y., Handley S.A., Baldridge M.T. (2021). The dark side of the gut: Virome-host interactions in intestinal homeostasis and disease. J Exp Med.

[53] Hsu C.L., Duan Y., Fouts D.E., Schnabl B. (2021). Intestinal virome and therapeutic potential of bacteriophages in liver disease. J Hepatol.

[54] Antoniak CE (1974). Mixtures of Dirichlet processes with applications to Bayesian nonparametric problems. Ann Statist.

[55] Ruzicka M (1958). Awndung matematisch-statishticher methoden in der geobotanik (syshetische bearbutung von autnahme). Biologia, Bratislava.

[56] Anthony Simon J (2015). Non-random patterns in viral diversity. Non-random patterns in viral diversity.

[57] Sommers Pacifica, Chatterjee Anushila, Varsani Arvind, Trubl Gareth (2021). Integrating Viral Metagenomics into an Ecological Framework. Annual Review of Virology.

[58] Zuo Tao (2020). Human-Gut-DNA Virome Variations across Geography, Ethnicity, and Urbanization. Cell Host Microbe.

[59] Szafrański Szymon P, Slots Jørgen, Stiesch Meike (2021). The human oral phageome. Periodontology 2000.

