# Supplementary material for: Stochastic neutral drifts seem prevalent in driving human virome assembly: Neutral, near-neutral and non-neutral theoretic analyses
Source: Comput Struct Biotechnol J. 2022 Mar 30;20:2029–41. doi: 10.1016/j.csbj.2022.03.027 (PMC9065738; doi:10.1016/j.csbj.2022.03.027)
Supplement: Supplementary Data 1 [file mmc1.pdf]

Online Supplementary Information for: Ma ZS & Mei JD (2022) Stochastic neutral drifts seem prevalent in driving human virome assembly: neutral, near-neutral and non-neutral theoretic analyses. *Computational and Structural Biotechnology Journal*.

Table S1. Brief information on the virome datasets used in this study, the datasets were processed uniformly with VirusSeeker virome pipeline (Zhao *et al*. 2017) to obtain the OTU tables

Dataset No.	NCBI Data Accession No.	Treatment	Sample Size	Virome Site Sampled	Research Focus in the Original Study	Reference
Dataset #1	SRP124915	Urban A	20	Feces	1. Viral classification<br>2. Phylogenetics	Siqueira JD, *et al.* (2018) Nature Communications
		Village B	10			
		Village C	16			
		Village D	15			
Dataset #2	SRP110665	Oct-2013	3	Feces	1. Meta-analyses<br>2. Relative abundance<br>3. KEGG pathways	Smits SA, *et al.* (2017) Science
		Jan-2014	8			
		Apr-2014	7			
		Aug-2014	12			
Dataset #3	SRP109620	Blood-Control LTR	16	Blood serum	1. Viral communities<br>2. Virus classification	Abbas AA, e*t al.* (2017) Am J Transplant
		Lung-Control-LTR	36	Bronchoalveolar Lavage		
		Lung-Control-OD	31			
Dataset #4	PRJNA421331	Healthy	5	Colon	1. Complexity and abundance<br>2.Three-dimensional PCA analysis	Wang *et al.* (2015) Inflammatory Bowel Disease
Total			179			

*LTR=lung transplant recipient; OD=Organ Donor

Table S2. The results of fitting Hubbell's UNTB (each sample is fitted with a standard single-site neutral model)

Datasets	Group	ID	*J*	*S*	*θ*	*m*	Observed	Predicted	95 % confidence	*P*
Dataset #1	Urban A	SRR6287103	20431	234	36.992	1.000	-247.120	-292	-293.886	1.000
		SRR6287104	38826	282	40.923	0.999	-296.053	-387	-389.219	1.000
		SRR6287105	25161	379	63.231	1.000	-303.754	-348	-348.577	1.000
		SRR6287106	575	58	15.915	1.000	-53.767	-47	-47.734	0.010
		SRR6287107	36192	286	41.676	0.991	-295.699	-379	-381.280	1.000
		SRR6287108	47065	193	25.602	1.000	-267.625	-358	-360.417	1.000
		SRR6287109	23654	216	32.601	0.999	-238.343	-301	-302.668	1.000
		SRR6287110	14538	77	10.559	0.999	-124.725	-175	-176.895	1.000
		SRR6287113	6034	193	37.862	1.000	-155.533	-170	-170.568	1.000
		SRR6287114	76225	222	28.261	0.991	-263.722	-433	-436.000	1.000
		SRR6287119	3302	57	9.662	1.000	-86.589	-101	-102.019	0.990
		SRR6287120	16189	182	28.757	1.000	-203.952	-252	-253.053	1.000
		SRR6287124	28158	165	23.161	1.000	-211.074	-294	-295.983	1.000
		SRR6287125	15098	124	18.428	0.990	-164.268	-216	-217.487	1.000
		SRR6287126	30390	79	9.852	0.993	-147.346	-213	-215.534	1.000
		SRR6287127	4153	58	9.399	0.996	-77.237	-108	-109.557	1.000
		SRR6287128	53391	170	21.588	0.991	-250.603	-352	-355.562	1.000
		SRR6287129	12291	248	43.968	1.000	-208.281	-240	-241.390	1.000
		SRR6287130	27927	137	18.674	1.000	-187.947	-268	-270.021	1.000
		SRR6287131	6420	152	27.840	0.980	-156.231	-168	-168.882	0.990
	Village B	SRR6287092	10726	225	40.278	1.000	-219.128	-224	-224.359	0.790

		SRR6287093	476	41	10.572	1.000	-39.235	-42	-42.449	0.920
		SRR6287095	21949	336	56.119	0.997	-291.582	-323	-324.555	1.000
		SRR6287096	12567	302	55.484	0.999	-245.828	-249	-249.844	0.750
		SRR6287097	23690	301	48.416	0.999	-277.124	-328	-328.748	1.000
		SRR6287098	1023	84	21.498	0.999	-76.470	-66	-66.245	0.000
		SRR6287099	18176	336	58.304	0.998	-265.312	-297	-297.445	1.000
		SRR6287100	2055	97	21.004	0.998	-97.591	-95	-95.692	0.190
		SRR6287101	14823	240	40.697	1.000	-232.995	-259	-260.471	1.000
		SRR6287102	22608	143	20.384	1.000	-192.908	-258	-259.782	1.000
		SRR6287076	9216	113	18.170	0.990	-153.005	-180	-181.405	1.000
		SRR6287077	13892	121	18.118	0.989	-161.590	-209	-211.175	1.000
		SRR6287078	267305	238	25.322	0.980	-373.114	-598	-603.034	1.000
		SRR6287080	211456	112	11.377	0.975	-211.342	-380	-384.914	1.000
		SRR6287081	109061	313	39.267	0.992	-379.380	-556	-559.761	1.000
		SRR6287082	9091	243	45.893	1.000	-193.441	-210	-210.849	1.000
		SRR6287083	11224	74	10.495	1.000	-113.297	-160	-161.078	1.000
	Village C	SRR6287084	23845	334	54.914	1.000	-300.281	-334	-335.379	1.000
		SRR6287086	35386	360	55.844	0.994	-337.942	-397	-398.047	1.000
		SRR6287111	9990	196	34.499	1.000	-186.387	-212	-212.632	1.000
		SRR6287112	36308	360	55.661	0.996	-336.939	-404	-405.106	1.000
		SRR6287115	69020	375	52.342	0.996	-397.387	-516	-518.270	1.000
		SRR6287116	54869	311	43.823	0.998	-333.638	-448	-449.979	1.000
		SRR6287117	28283	344	54.813	0.996	-324.929	-361	-361.962	1.000
		SRR6287118	4001	79	13.811	1.000	-110.083	-120	-121.497	0.960
		SRR6287134	41821	435	67.581	0.997	-375.028	-441	-442.657	1.000
		SRR6287074	368	16	3.267	1.000	-27.784	-30	-30.238	0.700
		SRR6287075	4755	246	54.830	1.000	-163.623	-149	-150.010	0.000
		SRR6287085	103163	212	25.778	0.996	-346.367	-462	-466.044	1.000
		SRR6287087	29803	339	53.283	0.999	-314.358	-367	-368.745	1.000
		SRR6287088	24461	367	60.856	0.999	-302.961	-343	-343.768	1.000
		SRR6287089	12343	207	35.134	1.000	-225.624	-232	-233.334	0.830
		SRR6287090	33892	322	49.225	1.000	-306.594	-382	-383.932	1.000
	Village D	SRR6287091	45219	312	45.176	1.000	-325.962	-419	-420.626	1.000
		SRR6287094	29150	274	41.583	1.000	-269.319	-348	-349.196	1.000
		SRR6287121	13208	379	72.874	1.000	-256.857	-257	-257.512	0.490
		SRR6287122	23268	258	40.560	1.000	-250.884	-314	-315.148	1.000
		SRR6287123	2122	149	36.301	0.999	-122.186	-97	-97.802	0.000
		SRR6287132	35686	404	64.094	0.919	-337.731	-410	-411.267	1.000
		SRR6287133	38792	418	65.774	0.978	-351.905	-424	-425.739	1.000
		SRR6287135	9567	168	28.896	1.000	-198.303	-204	-204.892	0.790
Dataset #2		SRR5763459	101312	533	76.424	0.665	-525.580	-651	-653.314	1.000
	Oct-2013	SRR5763462	74168	514	78.494	0.570	-465.404	-576	-578.064	1.000
		SRR5763463	74483	523	78.920	0.659	-486.909	-578	-580.086	1.000
		SRR5763457	117557	540	75.826	0.677	-552.912	-692	-694.958	1.000
		SRR5763458	66122	523	77.703	0.923	-448.273	-552	-553.265	1.000
		SRR5763475	115733	560	80.592	0.567	-568.677	-694	-696.398	1.000
	Jan-2014	SRR5763476	108881	578	83.117	0.681	-550.797	-684	-685.661	1.000
		SRR5763478	61526	507	75.393	0.988	-431.608	-531	-532.425	1.000
		SRR5763481	80973	517	75.377	0.800	-491.118	-594	-595.404	1.000
		SRR5763482	91603	571	82.453	0.878	-509.134	-636	-637.826	1.000
		SRR5763483	103416	556	76.743	0.984	-535.062	-660	-662.050	1.000
	Aug-2014	SRR5763445	97934	549	78.543	0.767	-521.209	-646	-648.366	1.000
		SRR5763448	97925	555	78.886	0.877	-531.348	-648	-650.688	1.000
		SRR5763456	67683	513	75.962	0.901	-452.664	-552	-553.501	1.000
		SRR5763467	62317	531	81.728	0.722	-451.359	-539	-540.668	1.000
		SRR5763468	49545	498	78.548	0.805	-409.450	-487	-488.830	1.000

		SRR5763469	107044	547	79.262	0.571	-548.919	-672	-674.426	1.000
		SRR5763470	55102	505	78.258	0.764	-427.613	-509	-510.402	1.000
		SRR5763471	94668	554	83.459	0.497	-530.180	-647	-649.222	1.000
		SRR5763472	80474	543	80.532	0.722	-491.435	-602	-603.896	1.000
		SRR5763473	72479	533	79.841	0.804	-476.108	-576	-577.616	1.000
Dataset #3	Blood-Control-LTR	SRR5710165	3218	70	12.520	1.000	-94.512	-108	-108.945	0.990
		SRR5710167	408	71	24.654	1.000	-47.023	-37	-37.379	0.000
		SRR5710170	118	5	0.905	0.999	-7.475	-11	-11.807	0.860
		SRR5710171	21088	17	1.761	0.999	-49.150	-73	-75.665	0.950
		SRR5710172	128	6	1.150	1.000	-8.634	-13	-13.213	0.880
		SRR5710176	1334	19	3.040	1.000	-34.847	-46	-46.909	0.940
		SRR5710178	602	15	2.809	0.640	-29.032	-33	-33.904	0.780
		SRR5710179	362	13	2.507	1.000	-21.762	-27	-27.457	0.890
		SRR5710180	322	3	0.340	1.000	-4.922	-9	-9.846	0.878
		SRR5710181	208	46	18.019	1.000	-46.294	-25	-24.955	0.000
		SRR5710183	1417	12	1.706	1.000	-24.375	-35	-36.742	0.940
		SRR5710186	664	9	1.359	0.966	-18.573	-24	-24.994	0.760
		SRR5710191	342	8	1.341	1.000	-14.555	-20	-20.738	0.870
	Lung-Control-LTR	SRR5826641	100	7	2.883	0.095	-22.043	-14	-14.089	0.000
		SRR5826642	360	28	10.935	0.157	-75.367	-36	-36.294	0.000
		SRR5826646	25203	284	52.800	0.239	-544.892	-340	-340.801	0.000
		SRR5826647	256	12	2.928	0.352	-30.113	-24	-24.075	0.020
		SRR5826648	1645	101	31.150	0.248	-200.053	-85	-85.462	0.000
		SRR5826653	180	21	9.948	0.184	-42.045	-24	-24.315	0.000
		SRR5826657	106	33	16.044	1.000	-21.744	-16	-16.187	0.000
		SRR5826659	998	109	30.967	1.000	-85.906	-64	-64.108	0.000
		SRR5826666	666	15	2.909	0.410	-33.816	-36	-36.946	0.680
		SRR5826676	10378	311	60.387	0.999	-216.513	-227	-227.609	0.990
		SRR5826679	2482	199	50.817	1.000	-120.612	-104	-104.487	0.000
		SRR5826681	239	24	6.462	1.000	-27.464	-28	-28.667	0.710
		SRR5826683	744	29	5.866	1.000	-43.110	-47	-47.502	0.830
		SRR5826689	852	20	3.573	0.962	-38.796	-43	-43.724	0.780
		SRR5826721	571	17	3.175	1.000	-33.436	-35	-35.527	0.590
		SRR5826724	201	12	2.643	1.000	-20.084	-21	-21.129	0.540
		SRR5826727	186	10	7.098	0.030	-20.019	-20	-20.206	0.470
		SRR5826760	200	9	7.001	0.021	-19.775	-19	-19.666	0.440
		SRR5826791	6749	279	58.483	1.000	-178.520	-181	-181.622	0.690
		SRR5826792	16544	342	60.972	0.998	-251.631	-285	-285.833	1.000
		SRR5826794	215	67	33.034	1.000	-42.740	-22	-22.762	0.000
		SRR5826795	959	131	40.815	1.000	-77.420	-60	-60.084	0.000
		SRR5826797	2268	190	49.264	0.999	-123.720	-99	-99.678	0.000
	Lung-Control-OD	SRR5826691	9735	295	57.215	0.999	-207.591	-219	-219.878	1.000
		SRR5826695	184	49	21.502	1.000	-27.003	-22	-22.319	0.000
		SRR5826701	5389	250	54.057	1.000	-173.288	-161	-161.649	0.010
		SRR5826706	416	90	35.012	1.000	-52.122	-36	-35.949	0.000
		SRR5826710	790	117	37.797	0.991	-79.264	-54	-54.042	0.000
		SRR5826714	792	16	2.814	0.657	-33.601	-38	-38.875	0.800
		SRR5826716	555	13	2.250	0.996	-26.590	-30	-31.127	0.790
		SRR5826718	298	22	5.322	1.000	-27.783	-30	-30.744	0.830
		SRR5826731	4562	208	44.742	1.000	-157.309	-148	-148.578	0.010
		SRR5826732	11613	322	61.159	1.000	-229.615	-240	-240.945	1.000
		SRR5826744	295	12	2.371	1.000	-25.265	-23	-24.150	0.410
		SRR5826747	1538	13	1.860	0.988	-31.092	-39	-39.983	0.860
		SRR5826749	1209	82	19.705	1.000	-69.445	-72	-72.198	0.810
		SRR5826768	2091	72	14.321	1.000	-80.041	-91	-91.522	1.000
		SRR5826771	2316	184	46.857	0.999	-119.521	-101	-101.211	0.000

		SRR5826772	2795	218	55.156	1.000	-126.813	-111	-111.185	0.000
		SRR5826779	207	53	22.705	1.000	-30.589	-24	-23.994	0.010
		SRR5826783	2095	75	15.098	1.000	-86.531	-92	-92.448	0.850
Dataset #4	Healthy	SRR6354727	8086	163	28.984	0.933	-174.493	-188	-189.045	1.000
		SRR6354728	2051	25	3.905	1.000	-38.065	-62	-63.230	1.000
		SRR6354729	598	31	6.787	1.000	-40.996	-45	-45.522	0.920
		SRR6354730	330	19	4.243	0.976	-27.363	-30	-30.875	0.850
		SRR6354731	107	12	3.266	1.000	-14.112	-16	-16.271	0.850

Table S3. The results of power analysis (non-neutral tests) and associated neutral tests (based on Hubbell's UNTB)

**Datasets**	***Groups***	***ID***	***J***	***S***	***θ***	***m***	***p-value***	***Ave. p* (IF)**	***Ave. p* (PC)**	***Power* (IF)**	***Power* (PC)**
Dataset #1	Urban A	SRR6287103	20431	234	36.992	1.000	1.00	0.53	0.53	0.00	0.00
		SRR6287104	38826	282	40.923	0.999	1.00	0.52	0.52	0.00	0.00
		SRR6287105	25161	379	63.231	1.000	1.00	0.55	0.55	0.02	0.02
		SRR6287106	575	58	15.915	1.000	0.01	0.46	0.46	0.10	0.10
		SRR6287107	36192	286	41.676	0.991	1.00	0.54	0.54	0.00	0.00
		SRR6287108	47065	193	25.602	1.000	1.00	0.54	0.54	0.00	0.00
		SRR6287109	23654	216	32.601	0.999	1.00	0.47	0.47	0.00	0.00
		SRR6287110	14538	77	10.559	0.999	1.00	0.50	0.50	0.00	0.00
		SRR6287113	6034	193	37.862	1.000	1.00	0.56	0.56	0.02	0.02
		SRR6287114	76225	222	28.261	0.991	1.00	0.51	0.51	0.00	0.00
		SRR6287119	3302	57	9.662	1.000	0.99	0.45	0.45	0.00	0.00
		SRR6287120	16189	182	28.757	1.000	1.00	0.51	0.51	0.00	0.00
		SRR6287124	28158	165	23.161	1.000	1.00	0.51	0.51	0.00	0.00
		SRR6287125	15098	124	18.428	0.990	1.00	0.48	0.48	0.00	0.00
		SRR6287126	30390	79	9.852	0.993	1.00	0.51	0.51	0.00	0.00
		SRR6287127	4153	58	9.399	0.996	1.00	0.47	0.47	0.00	0.00
		SRR6287128	53391	170	21.588	0.991	1.00	0.55	0.55	0.00	0.00
		SRR6287129	12291	248	43.968	1.000	1.00	0.54	0.54	0.00	0.00
		SRR6287130	27927	137	18.674	1.000	1.00	0.50	0.50	0.00	0.00
		SRR6287131	6420	152	27.84	0.980	0.99	0.48	0.48	0.02	0.02
		**Mean**	24301.000	175.600	27.248	0.996	0.950	0.509	0.509	0.008	0.008
		**Std. Err.**	4275.828	19.439	3.141	0.001	0.049	0.007	0.007	0.005	0.005
	Village B	SRR6287092	10726	225	40.278	1.000	0.79	0.50	0.50	0.00	0.00
		SRR6287093	476	41	10.572	1.000	0.92	0.41	0.41	0.02	0.02
		SRR6287095	21949	336	56.119	0.997	1.00	0.53	0.53	0.02	0.02
		SRR6287096	12567	302	55.484	0.999	0.75	0.51	0.51	0.06	0.06
		SRR6287097	23690	301	48.416	0.999	1.00	0.53	0.53	0.00	0.00
		SRR6287098	1023	84	21.498	0.999	0.00	0.52	0.52	0.04	0.04
		SRR6287099	18176	336	58.304	0.998	1.00	0.56	0.56	0.00	0.00
		SRR6287100	2055	97	21.004	0.998	0.19	0.54	0.54	0.02	0.02
		SRR6287101	14823	240	40.697	1.000	1.00	0.56	0.56	0.00	0.00
		SRR6287102	22608	143	20.384	1.000	1.00	0.50	0.50	0.00	0.00
		**Mean**	12809.300	210.500	37.276	0.999	0.765	0.516	0.516	0.016	0.016
		**Std. Err.**	2871.074	35.169	5.564	0.000	0.116	0.014	0.014	0.007	0.007
	Village C	SRR6287076	9216	113	18.17	0.990	1.00	0.49	0.49	0.00	0.00
		SRR6287077	13892	121	18.118	0.989	1.00	0.53	0.53	0.00	0.00
		SRR6287082	9091	243	45.893	1.000	1.00	0.51	0.51	0.02	0.02
		SRR6287083	11224	74	10.495	1.000	1.00	0.49	0.49	0.00	0.00
		SRR6287084	23845	334	54.914	1.000	1.00	0.59	0.59	0.00	0.00

		SRR6287086	35386	360	55.844	0.994	1.00	0.49	0.49	0.00	0.00
		SRR6287111	9990	196	34.499	1.000	1.00	0.57	0.57	0.04	0.04
		SRR6287112	36308	360	55.661	0.996	1.00	0.55	0.55	0.00	0.00
		SRR6287115	69020	375	52.342	0.996	1.00	0.55	0.55	0.00	0.00
		SRR6287116	54869	311	43.823	0.998	1.00	0.54	0.54	0.00	0.00
		SRR6287117	28283	344	54.813	0.996	1.00	0.52	0.52	0.00	0.00
		SRR6287118	4001	79	13.811	1.000	0.96	0.48	0.48	0.00	0.00
		SRR6287134	41821	435	67.581	0.997	1.00	0.54	0.54	0.00	0.00
		**Mean**	26688.154	257.308	40.459	0.997	0.997	0.527	0.527	0.005	0.005
		**Std. Err.**	5550.076	35.042	5.335	0.001	0.003	0.009	0.009	0.003	0.003
	Village D	SRR6287074	368	16	3.267	1.000	0.70	0.44	0.44	0.00	0.00
		SRR6287075	4755	246	54.83	1.000	0.00	0.55	0.55	0.02	0.02
		SRR6287087	29803	339	53.283	0.999	1.00	0.54	0.54	0.00	0.00
		SRR6287088	24461	367	60.856	0.999	1.00	0.60	0.60	0.00	0.00
		SRR6287089	12343	207	35.134	1.000	0.83	0.50	0.50	0.00	0.00
		SRR6287090	33892	322	49.225	1.000	1.00	0.57	0.57	0.00	0.00
		SRR6287091	45219	312	45.176	1.000	1.00	0.50	0.50	0.00	0.00
		SRR6287094	29150	274	41.583	1.000	1.00	0.52	0.52	0.00	0.00
		SRR6287121	13208	379	72.874	1.000	0.49	0.56	0.56	0.00	0.00
		SRR6287122	23268	258	40.56	1.000	1.00	0.54	0.54	0.02	0.02
		SRR6287123	2122	149	36.301	0.999	0.00	0.44	0.44	0.04	0.04
		SRR6287132	35686	404	64.094	0.919	1.00	0.56	0.56	0.00	0.00
		SRR6287133	38792	418	65.774	0.978	1.00	0.55	0.55	0.02	0.02
		SRR6287135	9567	168	28.896	1.000	0.79	0.54	0.54	0.00	0.00
		**Mean**	21616.714	275.643	46.561	0.992	0.772	0.529	0.529	0.007	0.007
		**Std. Err.**	3889.293	30.037	4.792	0.006	0.097	0.012	0.012	0.003	0.003
	**Mean**		22170.053	224.930	36.764	0.996	0.884	0.519	0.519	0.008	0.008
	**Std. Err.**		2272.505	14.925	2.407	0.002	0.037	0.005	0.005	0.002	0.002
Dataset #2	Blood-Control-LTR	SRR5710165	3218	70	12.52	1.000	0.99	0.43	0.43	0.00	0.00
		SRR5710167	408	71	24.654	1.000	0.00	0.55	0.55	0.04	0.04
		SRR5710170	118	5	0.905	0.999	0.86	0.43	0.43	0.00	0.00
		SRR5710171	21088	17	1.761	0.999	0.95	0.47	0.47	0.00	0.00
		SRR5710172	128	6	1.15	1.000	0.88	0.45	0.45	0.00	0.00
		SRR5710176	1334	19	3.04	1.000	0.94	0.45	0.45	0.00	0.00
		SRR5710178	602	15	2.809	0.640	0.78	0.40	0.40	0.00	0.00
		SRR5710179	362	13	2.507	1.000	0.89	0.44	0.44	0.00	0.00
		SRR5710180	322	3	0.34	1.000	0.88	0.43	0.43	0.00	0.00
		SRR5710181	208	46	18.019	1.000	0.00	0.49	0.49	0.08	0.08
		SRR5710183	1417	12	1.706	1.000	0.94	0.49	0.49	0.00	0.00
		SRR5710186	664	9	1.359	0.966	0.76	0.46	0.46	0.00	0.00
		SRR5710191	342	8	1.341	1.000	0.87	0.44	0.44	0.00	0.00
		**Mean**	2323.923	22.615	5.547	0.970	0.749	0.456	0.456	0.009	0.009
		**Std. Err.**	1581.207	6.608	2.155	0.028	0.094	0.010	0.010	0.007	0.007
	Lung-Control-LTR	SRR5826641	100	7	2.883	0.095	0.00	0.16	0.16	0.10	0.10
		SRR5826642	360	28	10.935	0.157	0.00	0.15	0.15	0.34	0.34
		SRR5826646	25203	284	52.8	0.239	0.00	0.30	0.30	0.06	0.06
		SRR5826647	256	12	2.928	0.352	0.02	0.32	0.32	0.00	0.00
		SRR5826648	1645	101	31.15	0.248	0.00	0.37	0.37	0.14	0.14
		SRR5826653	180	21	9.948	0.184	0.00	0.40	0.26	0.04	0.26
		SRR5826657	106	33	16.044	1.000	0.00	0.63	0.59	0.02	0.04
		SRR5826659	998	109	30.967	1.000	0.00	0.45	0.59	0.12	0.02
		SRR5826666	666	15	2.909	0.410	0.68	0.41	0.35	0.00	0.00

		SRR5826676	10378	311	60.387	0.999	0.99	0.50	0.64	0.04	0.00
		SRR5826679	2482	199	50.817	1.000	0.00	0.60	0.55	0.02	0.08
		SRR5826681	239	24	6.462	1.000	0.71	0.40	0.45	0.08	0.04
		SRR5826683	744	29	5.866	1.000	0.83	0.44	0.45	0.00	0.00
		SRR5826689	852	20	3.573	0.962	0.78	0.46	0.43	0.00	0.00
		SRR5826721	571	17	3.175	1.000	0.59	0.46	0.43	0.00	0.00
		SRR5826724	201	12	2.643	1.000	0.54	0.46	0.44	0.00	0.00
		SRR5826727	186	10	7.098	0.030	0.47	0.69	0.07	0.00	0.48
		SRR5826760	200	9	7.001	0.021	0.44	0.68	0.06	0.00	0.46
		SRR5826791	6749	279	58.483	1.000	0.69	0.53	0.55	0.02	0.06
		SRR5826792	16544	342	60.972	0.998	1.00	0.51	0.54	0.00	0.06
		SRR5826794	215	67	33.034	1.000	0.00	0.57	0.58	0.04	0.06
		SRR5826795	959	131	40.815	1.000	0.00	0.58	0.60	0.04	0.02
		SRR5826797	2268	190	49.264	0.999	0.00	0.56	0.54	0.08	0.10
		**Mean**	3134.870	97.826	23.920	0.682	0.337	0.462	0.410	0.050	0.101
		**Std. Err.**	1299.558	23.383	4.623	0.085	0.079	0.030	0.037	0.016	0.030
	Lung-Control-OD	SRR5826691	9735	295	57.215	0.999	1.00	0.49	0.52	0.04	0.04
		SRR5826695	184	49	21.502	1.000	0.00	0.55	0.61	0.06	0.04
		SRR5826701	5389	250	54.057	1.000	0.01	0.58	0.60	0.04	0.00
		SRR5826706	416	90	35.012	1.000	0.00	0.60	0.63	0.02	0.04
		SRR5826710	790	117	37.797	0.991	0.00	0.61	0.58	0.06	0.02
		SRR5826714	792	16	2.814	0.657	0.80	0.43	0.43	0.00	0.00
		SRR5826716	555	13	2.25	0.996	0.79	0.45	0.44	0.00	0.00
		SRR5826718	298	22	5.322	1.000	0.83	0.40	0.43	0.00	0.02
		SRR5826731	4562	208	44.742	1.000	0.01	0.52	0.51	0.04	0.04
		SRR5826732	11613	322	61.159	1.000	1.00	0.56	0.56	0.02	0.00
		SRR5826744	295	12	2.371	1.000	0.41	0.47	0.47	0.00	0.00
		SRR5826747	1538	13	1.86	0.988	0.86	0.45	0.45	0.00	0.00
		SRR5826749	1209	82	19.705	1.000	0.81	0.46	0.43	0.06	0.06
		SRR5826768	2091	72	14.321	1.000	1.00	0.43	0.48	0.02	0.02
		SRR5826771	2316	184	46.857	0.999	0.00	0.57	0.61	0.06	0.04
		SRR5826772	2795	218	55.156	1.000	0.00	0.59	0.56	0.06	0.02
		SRR5826779	207	53	22.705	1.000	0.01	0.59	0.57	0.02	0.06
		SRR5826783	2095	75	15.098	1.000	0.85	0.51	0.45	0.02	0.04
		**Mean**	2604.444	116.167	27.775	0.979	0.466	0.514	0.518	0.029	0.024
		**Std. Err.**	777.834	24.301	5.047	0.019	0.104	0.016	0.017	0.006	0.005
	**Mean**		2762.833	85.833	20.782	0.851	0.479	0.478	0.457	0.033	0.053
	**Std. Err.**		708.309	13.685	2.863	0.042	0.057	0.014	0.018	0.007	0.014
Dataset #3	Healthy	SRR6354727	8086	163	28.984	0.933	1.00	0.53	0.51	0.02	0.00
		SRR6354728	2051	25	3.905	1.000	1.00	0.45	0.44	0.00	0.00
		SRR6354729	598	31	6.787	1.000	0.92	0.44	0.43	0.00	0.00
		SRR6354730	330	19	4.243	0.976	0.85	0.44	0.45	0.02	0.00
		SRR6354731	107	12	3.266	1.000	0.85	0.41	0.44	0.06	0.02
	**Mean**		2234.400	50.000	9.437	0.982	0.924	0.454	0.454	0.020	0.004
	**Std. Err.**		1501.753	28.425	4.923	0.013	0.034	0.020	0.014	0.011	0.004
**Mean**			12276.362	152.638	28.146	0.928	0.697	0.497	0.488	0.020	0.029
**Std. Err.**			1472.802	11.791	1.962	0.021	0.037	0.007	0.009	0.004	0.007

Table S4. The mean parameters from the neutral tests and non-neutral tests (power analysis) for the virome datasets, summarized from Table S3

Datasets	*Groups*	*ID*	*J*	*S*	*θ*	*m*	*p-value*	*Ave. p* (IF)	*Ave. p* (PC)	*power* (IF)	*power* (PC)
Dataset #1	Urban A	**Mean**	24301.000	175.600	27.248	0.996	0.950	0.509	0.509	0.008	0.008
		**Std. Err.**	4275.828	19.439	3.141	0.001	0.049	0.007	0.007	0.005	0.005
	Village B	**Mean**	12809.300	210.500	37.276	0.999	0.765	0.516	0.516	0.016	0.016
		**Std. Err.**	2871.074	35.169	5.564	0.000	0.116	0.014	0.014	0.007	0.007
	Village C	**Mean**	26688.154	257.308	40.459	0.997	0.997	0.527	0.527	0.005	0.005
		**Std. Err.**	5550.076	35.042	5.335	0.001	0.003	0.009	0.009	0.003	0.003
	Village D	**Mean**	21616.714	275.643	46.561	0.992	0.772	0.529	0.529	0.007	0.007
		**Std. Err.**	3889.293	30.037	4.792	0.006	0.097	0.012	0.012	0.003	0.003
Dataset #2	Blood-Control-LTR	**Mean**	2323.923	22.615	5.547	0.970	0.749	0.456	0.456	0.009	0.009
		**Std. Err.**	1581.207	6.608	2.155	0.028	0.094	0.010	0.010	0.007	0.007
	Lung-Control-LTR	**Mean**	3134.870	97.826	23.920	0.682	0.337	0.462	0.410	0.050	0.101
		**Std. Err.**	1299.558	23.383	4.623	0.085	0.079	0.030	0.037	0.016	0.030
	Lung-Control-OD	**Mean**	2604.444	116.167	27.775	0.979	0.466	0.514	0.518	0.029	0.024
		**Std. Err.**	777.834	24.301	5.047	0.019	0.104	0.016	0.017	0.006	0.005
Dataset #3	Healthy	**Mean**	2234.400	50.000	9.437	0.982	0.924	0.454	0.454	0.020	0.004
		**Std. Err.**	1501.753	28.425	4.923	0.013	0.034	0.020	0.014	0.011	0.004

Table S5. Fitting to Sloan's (2006, 2007) neutral model (SNM) with the human virome datasets, with both source and destination communities being the same.

**Datasets**	**Source Community**	**Destination Community**	***N***	**Immigration Probability (*m*)**	R^2	**Total Number of Species**	**Percentage of Species Below Neutral (%)**	Percentage of Neutral Species (%)	**Percentage of Species Above Neutral (%)**
Dataset #1	Urban A	Urban A	24301	0.037	0.266	634	12.1	71.3	16.6
	Village B	Village B	12809	0.111	0.367	556	8.3	86.2	5.6
	Village C	Village C	58423	0.062	0.338	689	12.5	73.7	13.8
	Village D	Village D	27053	0.107	0.526	700	9.7	83.6	6.7
Dataset #2	Oct-2013	Oct-2013	83321	1.586	0.620	604	0.0	75.0	25.0
	Jan-2014	Jan-2014	93226	1.196	0.817	699	1.3	80.3	18.5
	Apr-2014	Apr-2014	266707	0.735	0.773	718	2.2	78.4	19.4
	Aug-2014	Aug-2014	96039	0.895	0.871	714	1.7	77.3	21.0
Dataset #3	Blood-Control-LTR	Blood-Control-LTR	1896	0.023	0.120	169	3.0	94.1	3.0
**Mean**			73753	0.528	0.522	609	5.6	80.0	14.4
**Standard Error**			26858	0.197	0.089	58	1.7	2.4	2.6

Table S6. Comparison of the assignment results from VirusSeeker *vs*. Genome Detective*

**Sample**		**VirusSeeker**		**Genome Detective**		
**Sample_ID**	**Cohort (Treatment)**	**Num. of Assignment (Species)**	**Num. of Reads**	**Num. of Assignment (Species)**	**Num. of Reads**	**Num. of Contigs**
SRR6354717	CD	93	637	8	102	24
SRR6354718	CD	155	1026	4	655	25
SRR6354719	CD	112	410	5	127	30
SRR6354720	CD	2	40	4	403	24
SRR6354721	CD	131	869	10	1032	94

SRR6354722	CD	102	596	4	334	28
SRR6354723	UC	102	500	2	109	11
SRR6354724	UC	61	251	7	313	112
SRR6354725	UC	122	621	6	410	34
SRR6354726	UC	180	1070	10	1357	215
SRR6354727	Control	83	537	0	0	0
SRR6354728	Control	4	9	1	2	1
SRR6354729	Control	7	51	1	134	4
SRR6354730	Control	7	22	1	28	3
SRR6354731	Control	3	5	1	10	3

*Genome Detective Software can be downloaded from:
https://www.genomedetective.com/app/typingtool/virus/how-to-cite
